# Identification of a novel thermostable transaminase and its application in L-phosphinothricin biosynthesis

**DOI:** 10.1007/s00253-024-13023-7

**Published:** 2024-01-30

**Authors:** Han-Lin Liu, Pu-Hong Yi, Jia-Min Wu, Feng Cheng, Zhi-Qiang Liu, Li-Qun Jin, Ya-Ping Xue, Yu-Guo Zheng

**Affiliations:** 1 Joint Engineering Research Center for Biomanufacturing of Chiral Chemicals, The National and Local, Zhejiang University of Technology, Hangzhou, 310014 People’s Republic of China; 2 Engineering Research Center of Bioconversion and Biopurification of Ministry of Education, Zhejiang University of Technology, Hangzhou, 310014 People’s Republic of China; 3 Key Laboratory of Bioorganic Synthesis of Zhejiang Province, College of Biotechnology and Bioengineering, Zhejiang University of Technology, Hangzhou, 310014 People’s Republic of China

**Keywords:** Thermostable transaminase, L-phosphinothricin, Enzyme cascade, Asymmetric synthesis, Deracemization

## Abstract

**Abstract:**

Transaminase (TA) is a crucial biocatalyst for enantioselective production of the herbicide L-phosphinothricin (L-PPT). The use of enzymatic cascades has been shown to effectively overcome the unfavorable thermodynamic equilibrium of TA-catalyzed transamination reaction, also increasing demand for TA stability. In this work, a novel thermostable transaminase (*Pt*TA) from *Pseudomonas thermotolerans* was mined and characterized. The *Pt*TA showed a high specific activity (28.63 U/mg) towards 2‐oxo‐4‐[(hydroxy)(methyl)phosphinoyl]butyric acid (PPO), with excellent thermostability and substrate tolerance. Two cascade systems driven by *Pt*TA were developed for L-PPT biosynthesis, including asymmetric synthesis of L-PPT from PPO and deracemization of D, L-PPT. For the asymmetric synthesis of L-PPT from PPO, a three-enzyme cascade was constructed as a recombinant *Escherichia coli* (*E. coli* G), by co-expressing *Pt*TA, glutamate dehydrogenase (GluDH) and D-glucose dehydrogenase (GDH). Complete conversion of 400 mM PPO was achieved using only 40 mM amino donor L-glutamate. Furthermore, by coupling D-amino acid aminotransferase (*Ym* DAAT) from *Bacillus sp. YM‐1* and *Pt*TA, a two-transaminase cascade was developed for the one-pot deracemization of D, L-PPT. Under the highest reported substrate concentration (800 mM D, L-PPT), a 90.43% L-PPT yield was realized. The superior catalytic performance of the *Pt*TA-driven cascade demonstrated that the thermodynamic limitation was overcome, highlighting its application prospect for L-PPT biosynthesis.

**Key points:**

*• A novel thermostable transaminase was mined for L-phosphinothricin biosynthesis.*

*• The asymmetric synthesis of L-phosphinothricin was achieved via a three-enzyme cascade.*

*• Development of a two-transaminase cascade for D, L-phosphinothricin deracemization.*

**Supplementary Information:**

The online version contains supplementary material available at 10.1007/s00253-024-13023-7.

## Introduction

Phosphinothricin (2-amino-4-(hydroxy(methyl)phosphoryl)butyric acid, PPT) is a commercial broad-spectrum herbicide, and only L-enantiomer (L-PPT) has the herbicidal activity (Kang et al. 2019; Zhang et al. 2017; Zhao et al. 2023b). Therefore, it is necessary to synthesize optically pure L-PPT or increase the L-PPT content in racemic D, L-PPT (Takano and Dayan 2020). Biosynthetic methods have recently been developed and focused on, providing simple methods to produce optically pure L-isomer by asymmetric synthesis or deracemization (Cao et al. 2021; Cheng et al. 2022; Zhou et al. 2020). For efficient biosynthesis of L-PPT, the biocatalyst employed should be highly selective, tolerant in substrate loading, and operationally and thermally stable.

As a significant class of enzymes that take part in the biosynthesis of L-PPT, transaminases (EC 2.6.1.X, TAs) represent enzymes with pyridoxal-5′-phosphate (PLP) dependence, catalyzing reversible amino groups transfer between amino donor and acceptor (Guo and Berglund 2017; Mathew et al. 2023; Slabu et al. 2017). The TAs are divided into PLP fold types I and IV (Meng et al. 2021), and TAs that belong to fold type I only appear as (*S*)-enantioselective (Börner et al. 2017; Konia et al. 2021). The asymmetric synthesis of L-PPT from 4-(hydroxy(methyl)phosphoryl)-2-oxobutanoic acid (PPO) catalyzed by fold-type I TAs with 100% theoretical yield and strict (*S*)-enantioselectivity has continuously attracted the interest of researchers (Cheng et al. 2021; Horsman and Zechel 2017). However, several TAs developed for the asymmetric synthesis of L-PPT suffered low equilibrium constant, resulting in an unfavorable thermodynamic equilibrium, which further led to the inability to achieve the theoretical yield (Liu et al. 2023; Zhu and Hua 2009), for example, the TA from *Citrobacter koseri* (*Ck*TA), which showed a maximum conversion of 93.3% at 100 mM substrate concentration (Jia et al. 2019), and the TA from *Pseudomonas fluorescens* (*Pf*TA), which we previously reported to yield 79% L-PPT after 24 h reaction at 500 mM substrate (Jin et al. 2019). Removal of by-product can effectively shift the equilibrium in the transamination reaction, one of the most attractive strategies was to construct an enzymatic cascade by coupling amino acid dehydrogenases (AADHs, EC 1.4.1.X) (Hepworth et al. 2017; Mathew et al. 2023), such as alanine dehydrogenase (AlaDH, EC 1.4.1.1) or glutamate dehydrogenase (GluDH, EC 1.4.1.2) (Wu et al. 2023). The cascade system can regenerate amino donor with cofactor NAD(P)H, supplying amino groups through using cheap inorganic ammonia (Dave and Kadeppagari 2019). In addition, regeneration of the cofactor NAD(P)H could be achieved by glucose dehydrogenase (GDH, EC 1.1.1.47), alcohol dehydrogenase (ADH, EC 1.1.1.1), or formate dehydrogenase (FDH, EC 1.2.1.2) (Guo et al. 2020; Qian et al. 2020; Zan et al. 2023). On the other hand, the high stability of biocatalyst was needed, since each enzyme in a multi-enzyme system has its own enzymatic properties and the operating conditions of multi-enzyme systems typically adopted compromise parameters (Wang et al. 2021).

There are two main strategies, gene mining and protein engineering, to obtain novel biocatalysts (Guo and Berglund 2017). Mining and characterizing microorganisms isolated from extreme environments are an effective strategy for discovering novel enzymes that can withstand strict industrial standards, such as high temperatures and the presence of organic solvents (Cerioli et al. 2015; Kelly et al. 2020; Mathew et al. 2016). For example, this strategy was used to identify a thermostable TPTA*gth* from *Geobacillus thermodenitrificans*, which exhibited excellent thermal stability at a maximum temperature of 65 °C (Chen et al. 2016).

**Fig. 1:**
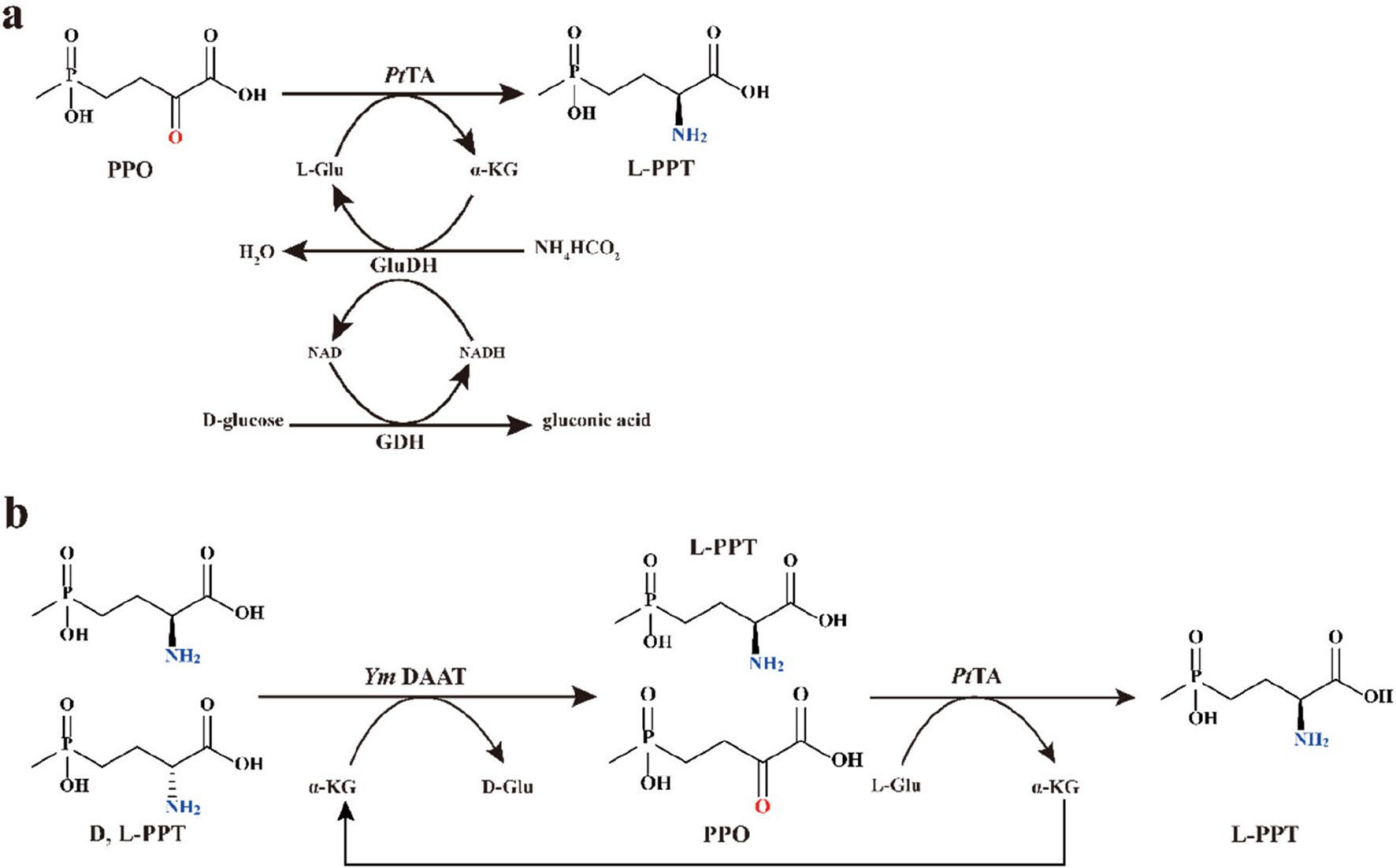
Enzyme cascade systems for biosynthesis of L-phosphinothricin. **a** Three-enzyme cascade coupling *Pt*TA, GluDH, and GDH for the asymmetric synthesis of L-PPT. **b** Two-transaminase cascade for deracemization of D, L-PPT. *Pt*TA, transaminase from *Pseudomonas thermotolerans*; GluDH, glutamate dehydrogenase; GDH, D-glucose dehydrogenase; *Ym* DAAT, D-amino acid aminotransferase from *Bacillus sp.* YM-1

At present, most of the TAs obtained for producing L-PPT are based on their catalytic activity towards PPO. Since substrate loading and enzyme stability directly affect production yields, these factors should be considered for the discovery of industrial biocatalysts (Xie et al. 2022). In this work, sequence mining and structure–function analysis were performed to obtain a thermostable TA from *Pseudomonas thermotolerans* (*Pt*TA) with high activity and stability. To evaluate its potential in practical applications, two cascade systems were developed using PPO and D, L-PPT as starting substrate, respectively (Fig. 1). By employing *Pt*TA-driven cascade systems, L-PPT biosynthesis was realized at high substrate concentrations, which demonstrated its potential effectiveness as an industrial biocatalyst.

## Materials and methods

### Strains and chemicals

The enzymes, plasmids, and primers are summarized in Table S1. D, L-PPT, and PPO were obtained from Shandong Lvba Chemical Co., Ltd. (Jinan, China). NAD^+^ and NADH were obtained from Roche Diagnostics GmbH (Mannheim, Germany). PLP and different L-amino acids were obtained from J&K Scientific Ltd. (Beijing, China). Other chemicals were analytical reagent and could be obtained commercially.

### Gene mining of *Pt*TA

The amino acid sequence of transaminase (*Se*TA, GenBank: WP_001095559.1) from *Salmonella enterica* was used as a probe for homology searching in the NCBI protein database (https://blast.ncbi.nlm.nih.gov/Blast.cgi). The amino acid sequences from different genera with 30–80% identity were selected from a pool of 1000 max target sequences. The neighbor-joining phylogenetic tree was generated using the MEGA 7.0 program, and bootstrap values were calculated from 1000 replicates.

### Substrate docking simulation

The protein model of *Pt*TA containing coenzyme pyridoxamine 5′-phosphate (PMP) was used as a receptor, and the PPO was used as a ligand. The ligand (PPO) was obtained by Chemdraw and Chem3D program, and energy minimization was performed using the Chem3D MM2 minimize tool. Molecule docking simulation was performed using AutoDock 4.2 (search parameters: Genetic Algorithm Parameters; Output: Lamarckian GA (4.2)). “The residues I50, Y138, R141, Y155, E211 and R398 were selected as docking sites, and the grid box for docking was set based on their spatial coordinate. Based on its substrate recognition mechanism and lowest binding energy, the docking model was selected.” Visualization of the homology model with docking simulation was performed by Pymol software.

### Cloning and expression

The *Pt*TA gene (GenBank: WP_017938159.1) was amplified by PCR using primers A-*Pt*TA-F and A-*Pt*TA-R with genomic DNA of *Pseudomonas thermotolerans J53* (taxid:935,867) as template under conditions: 95 °C for 5 min, 1 cycle; 95 °C for 30 s; 55 °C for 30 s; 72 °C for 90 s, 35 cycles; 72 °C for 10 min. After extraction and purification of PCR products, the plasmid pET-28a-*Pt*TA was constructed by in-fusion cloning. Then, it was transformed into *Escherichia coli* (*E. coli*) BL21(DE3) (Novagen, Germany) for expression. The recombinant *E. coli* was cultivated at 37 °C in 100-mL TB medium including 50 µg kanamycin for the OD_600_ reached 0.6–0.8. The 0.1 mM isopropyl-β-D-thiogalactoside (IPTG) was added to induce the expression at 28 °C for 12 h. The cells were harvested by centrifugation (8000 rpm, 4 °C for 10 min) and resuspended in 20 mM potassium phosphate buffer (PB buffer, pH 8.0). Sonication was performed to prepare crude cell extract in intermittent pulse mode (100 W, 1 s duration, 2-s interval for 15 min). The protein was purified by Nickel column affinity chromatography (Jin et al. 2019). SDS‐polyacrylamide gel electrophoresis (SDS‐PAGE) was used to analyze the expression of *Pt*TA.

### Enzyme activity assay of *Pt*TA

One unit (U) of *Pt*TA activity was defined as the quantity of protein needed for generating 1 µmol L-PPT per minute under the standard conditions. The standard conditions were performed in 1 mL PB buffer (50 mM, pH 8.0) at 55 °C for 10 min, including 0.1 mM PLP, 20 mM PPO, 100 mM L-amino acid, and purified enzyme.

### Characterization of *Pt*TA and kinetic parameters

Temperature effect on *Pt*TA activity was measured at 30–70 °C. For temperature stability, the residual activity was measured after purified *Pt*TA were incubated at 40–65 °C for 6 h in 50 mM PB buffer (pH 8.0) that contained 0.1 mM PLP. Using phosphate buffer (PB buffer, 50 mM, pH 6.0–8.0), Tris–HCl buffer (50 mM, pH 7.5–9.0), and Na_2_B_4_O_7_-NaOH buffer (50 mM, pH 9.0–10.0), pH effect on *Pt*TA activity was measured. The pH stability of *Pt*TA were measured through incubating purified protein in 4 °C buffers for 24 h.

To measure the kinetic parameters of *Pt*TA, the nonlinear fit of Michaelis–Menten model was employed. For the measurement of initial reaction rates, the reaction was performed under standard conditions, with the exception of using varying concentrations of PPO (1–100 mM). Additionally, the conversion of PPO was limited to 10%.

### Asymmetric synthesis of L-PPT from PPO via cascade system

Co-expressed recombinant *E. coli* (A-F) containing *Ls*GluDH and *Es*GDH and co-expressed recombinant *E. coli* G containing *Ls*GluDH, *Es*GDH, and *Se*TA were summarized in Table S2. Using a ribosome binding site (RBS) calculator to design the synthetic RBS sequence of EsGDH (https://www.denovodna.com/software/design_rbs_calculator) (Salis et al. 2009).

Asymmetric synthesis of L-PPT via *E. coli G* was conducted at 35–55 °C. Thirty-milliliter reaction system (pH 7.0–9.0) containing 300–500 mM PPO, 30–50 mM L-Glu, 360–600 mM (NH_4_)_2_SO_4_, 390–650 mM D-glucose, 0.1 mM PLP, 0.1 mM NAD^+^, and 4 g/L dry cell weight (DCW) *E. coli G.* The pH of 30 mL reaction system was controlled by an automatic pH titrator (Metrohm 902 Titrando) via the addition of ammonia.

### Deracemization of D, L-PPT

The deracemization of racemic PPT at high substrate concentration was detected in PB buffer (50 mM, pH 8.0) at 45 °C. Thirty-milliliter system including 3 g/L DCW *E. coli*/pET28a-*Pt*TA, 1.5 g/L DCW *E. coli*/pET28a-*Ym* DAAT, 200–800 mM D, L-PPT, 0.5–2 M L-Glu, 2–8 mM α-KG, and 0.2 mM PLP.

### Analytical methods

The Ultimate 3000 HPLC system (ThermoFisher, Dionex, USA) with a fluorescence detector (UltiMate FLD-3100) and a C18 column (Welchrom® C18, 4.6 mm × 250 mm, 5 µm; China) was used to measure the L-PPT and D-PPT concentrations. After derivatization of samples at 30 °C for 5 min, the quantitative chiral analyses were detected at fluorescence wavelengths of λex = 340 nm and λem = 450 nm. The chiral derivatization reagent was prepared by *O*-phthalaldehyde and *N*-acetyl-L-cysteine (Jin et al. 2019).

The detection of PPO concentration was carried out in the above HPLC system with a diode array detector (UltiMate DAD-3000) (Cao et al. 2021). Detection was performed at 232 nm with a mobile phase flow rate of 1.0 mL/min, and a ratio of 12:88 (v/v) of acetonitrile and 50 mM ammonium dihydrogen phosphate buffer was used as the mobile phase. The HPLC chromatograms of chemicals are shown in Fig. S1.

## Results

### Gene mining of thermostable transaminase

In order to obtain novel TAs with the desired function, transaminase *Se*TA, which possessed the ability to synthesize L-PPT from PPO (Jin et al. 2022), was chosen as a template for homologous sequence searching. Gene sequence screening and alignment were performed in the NCBI protein database, and the TA sequences with 30–80% identity were selected from different species for constructing a phylogenetic tree (Fig. S2). It was found that most of the selected TA sequences were from *Pseudomonas*, followed by *Klebsiella*, *Escherichia*, *Citrobacter*, *Shigella*, *Leclercia*, *Atopomonas*, etc. Among them, a transaminase (*Pt*TA) from *Pseudomonas thermotolerans* shared sequence identity of 71.7% to *Se*TA was identified, and the *Pseudomonas thermotolerans* was described as a thermotolerant species with a maximal growth temperature of 55 °C (Manaia and Moore 2002). The *Pt*TA was defined as 4-aminobutyrate-2-oxoglutarate transaminase (gamma-aminobutyrate transaminase, GABA-TA, EC 2.6.1.19) in the NCBI protein database with unreported enzymatic properties and application.

**Fig. 2:**
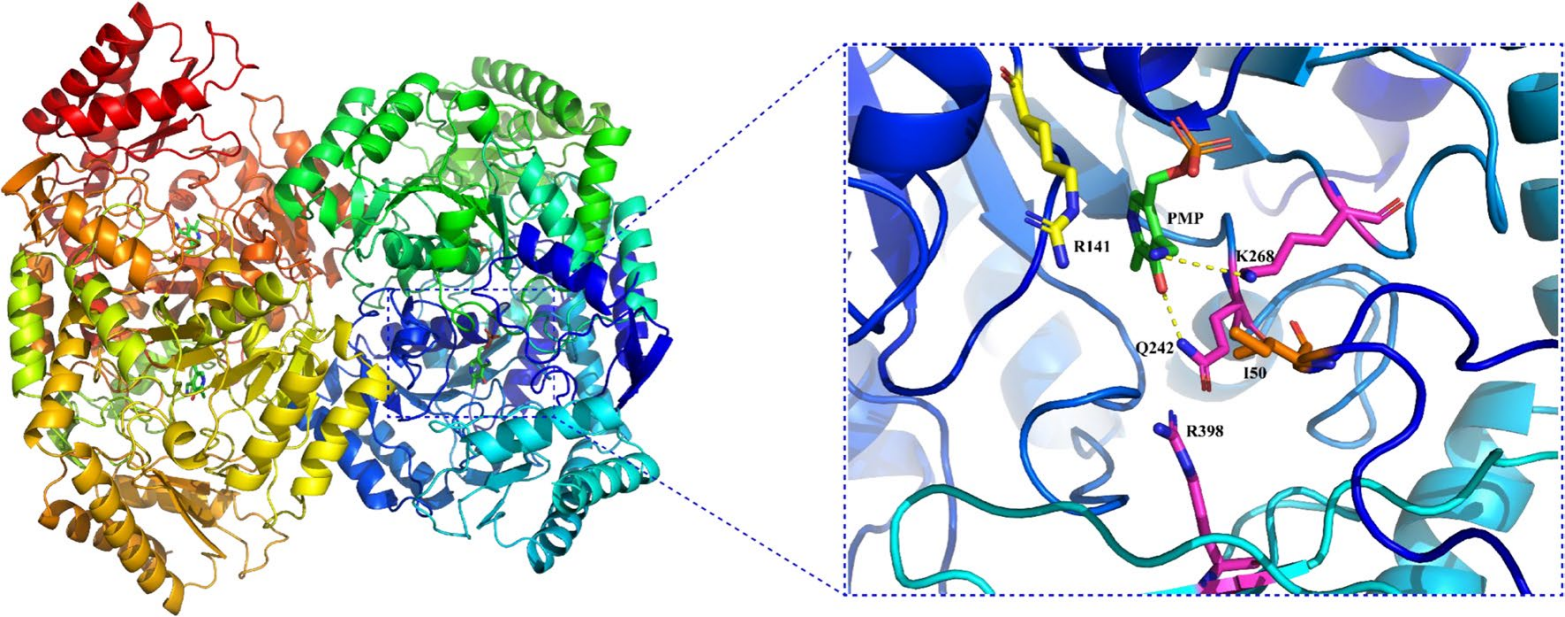
Homology model of *Pt*TA and key active site residues

According to the understanding of the structure and function relationship, identifying active site residues has been applied in function prediction (Jiang et al. 2023; Petermeier et al. 2023). Using a GABA-TA (PDB ID: 1SZK; amino acid sequence identity: 76.42%) as a template, the homology modeling was prepared by SWISS-MODEL (https://swissmodel.expasy.org/). The 3D model of the protein showed that *Pt*TA could have a homo-tetrameric structure, with the PMP docked to the center of the active site of each subunit, and three completely conserved residues (K268, Q242 and R398) were identified (Fig. 2). The model suggests that K268 is involved in forming a Schiff base with the cofactor PLP, while Q242 and R398 may be involved in coordinating the 3′-phenolic oxygen of PLP and substrate recognition, respectively (Liu et al. 2005). Compared with template *Se*TA, the I50 and R141 residues were found in *Pt*TA as well. The I50 residue has been reported to act as a hydrophobic lid that restricted substrate binding pocket size (Liu et al. 2004, 2005). Through substrate docking simulation using PPO as a ligand, the R141 residue formed a hydrogen bond with γ-phosphinoyl group of ligand PPO (Fig. S3), indicating that residue R141 residue plays an important role in stabilizing substrate PPO binding. The above results suggested that the *Pt*TA has a similar substrate binding pocket to *Se*TA (Fig. S4), and it is further postulated that *Pt*TA could accept PPO as a substrate.

**Fig. 3:**
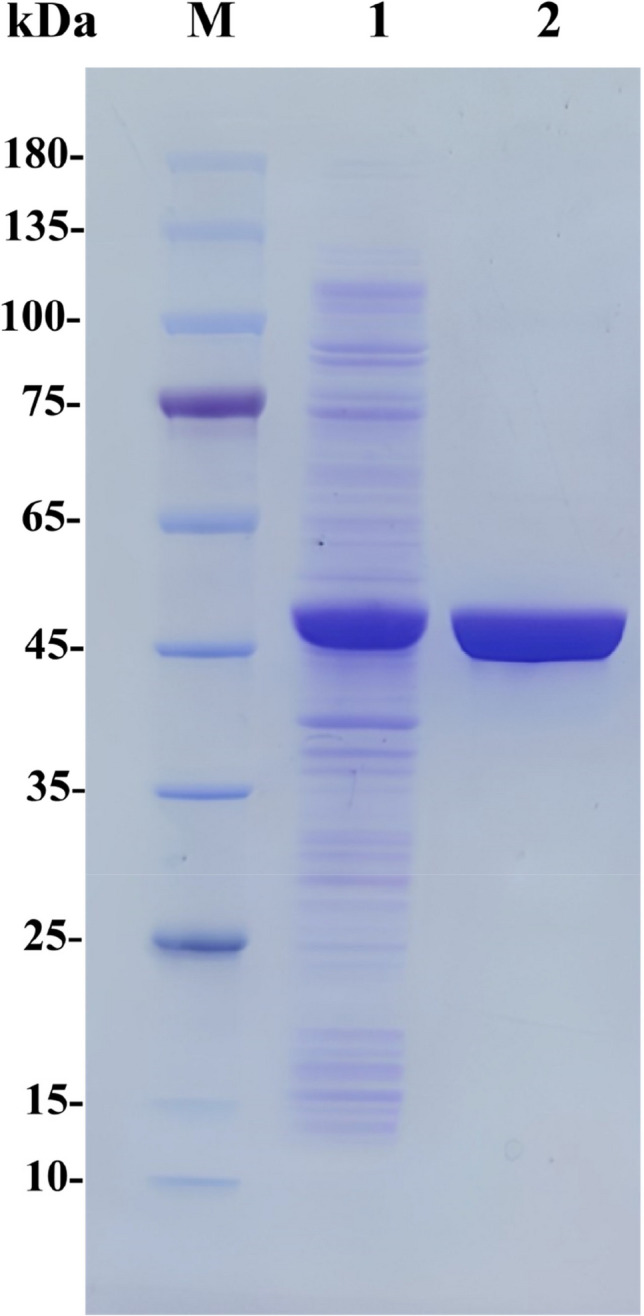
SDS-PAGE analysis of *Pt*TA. Lane M, protein marker; Lane 1, crude extract; Lane 2, purified *P*tTA. The protein molecular mass of *Pt*TA was 45.96 kDa

Therefore, the gene of *Pt*TA was cloned into plasmid pET28a and expressed in *E. coli* BL21(DE3). The overexpression and purification of *Pt*TA were analyzed by SDS-PAGE (Fig. 3), and the observed protein masses of around 45 kDa correspond well with the calculated masses of the His6-tagged *Pt*TA, which is 45.96 kDa. Using L-Ala as an amino donor, *Pt*TA exhibited a 1.62 U/mg specific activity towards PPO.

### Amino donor specificity of *Pt*TA

The specific activity of *Pt*TA towards PPO needs to be optimized to meet the application requirements, and we assumed that L-Ala might not be a suitable amino donor. Using various L-amino acids and isopropylamine (IPA) as the amino donors, the amino donor specificity of *Pt*TA was investigated by detecting the generation of L-PPT. As expected, *Pt*TA preferred L-Glu as an amino donor (28.63 U/mg), followed by L-Ala (Table 1). Among the remaining amino donors, the by-product oxaloacetic acid and acetone of L-aspartate (L-Asp) and IPA, respectively, can be easily removed via decarboxylation or evaporation, which can promote thermodynamic equilibrium towards product synthesis (Fang et al. 2020; Kelefiotis-Stratidakis et al. 2019). However, *Pt*TA was inactive towards IPA and L-Asp. Interestingly, the by-product of L-Glu deamination, α-KG, can be easily regenerated to L-Glu by GluDH, suggesting that the by-product inhibition can be overcome by employing a cascade system coupled with GluDH. Thus, amino donor L-Glu was chosen in this study.

**Table 1:** Amino donor specificity of *Pt*TA

Amino donor	Specific activity (U/mg)^a^	e.e. (%)^b^
L-glutamate	28.63 ± 0.31	> 99.9
L-alanine	1.62 ± 0.07	> 99.9
L-threonine	0.02 ± 0.08	> 99.9
L-phenylalanine	0.04 ± 0.02	> 99.9
L-valine	0.07 ± 0.09	> 99.9
L-isoleucine	0.14 ± 0.03	> 99.9
L-proline	0.08 ± 0.18	> 99.9
L-aspartate	0.05 ± 0.03	> 99.9
isopropylamine	0	-

^a^The specific activity was performed at standard assay conditions

^b^Enantiomeric excess (e.e.) was determined by chiral HPLC analysis. $e.e.=\frac{\left[{\text{L}}-{\text{PPT}}\right]-[{\text{D}}-{\text{PPT}}]}{\left[{\text{L}}-{\text{PPT}}\right]+[{\text{D}}-{\text{PPT}}]}\times 100\mathrm{\%}$; [L‐PPT] and [D‐PPT] represent the concentrations of L-PPT and D-PPT, respectively

### Characterization of *Pt*TA

**Fig. 4:**
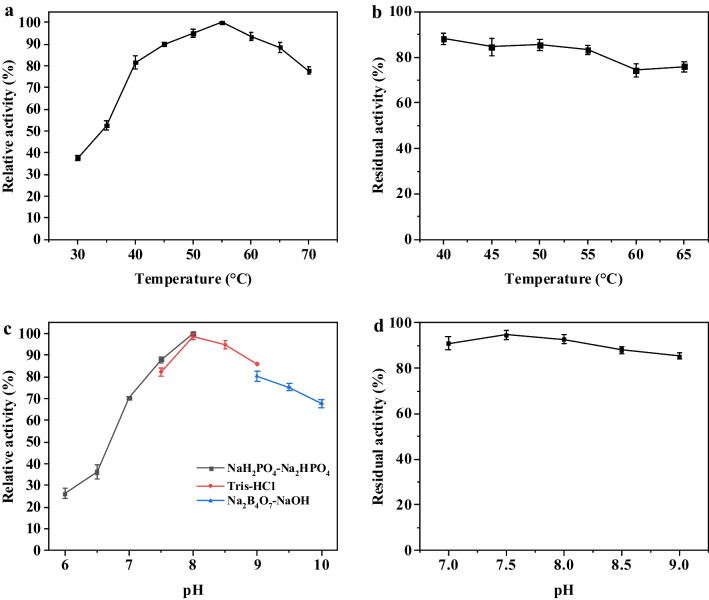
Characterization of *Pt*TA. **a** Effect of temperature on enzymatic activity. **b** Effect of temperature on thermostability. **c** Effect of pH on enzymatic activity. **d** Effect of pH on enzyme stability. The residual activity was defined to the percentage of measured activity to initial activity

To characterize the enzymatic properties of *Pt*TA, the effects of temperature and pH on activity and stability were detected using L-Glu and PPO as substrates. As shown in Fig. 4a, *Pt*TA exhibited the highest activity at a temperature of 55 °C. Of particular interest was that *Pt*TA showed a relative activity of more than 80% in the 40 to 65 °C range, indicating a preference for higher reaction temperatures and the capability to adapt to the temperature required in different enzyme cascades. Therefore, the evaluation of *Pt*TA for temperature stability was carried out by incubating purified *Pt*TA in PB buffer at 40–65 °C. *Pt*TA could retain 83.21% residual activity after incubation at 55 °C for 6 h (Fig. 4b), suggesting excellent thermostability.

The optimal pH of *Pt*TA was pH 8.0 using PB buffer, and more than 80% relative activity was observed between pH 7.5 and pH 9.0 (Fig. 4c). The *Pt*TA was stable between pH 7.0 and 9.0, it retained over 85% residual activity after incubated for 24 h (Fig. 4d). The substrate tolerance of *Pt*TA was investigated at PPO concentrations ranging from 20 to 600 mM (3.96–118.8 g L^−1^). The L-PPT yield still reached 53.48% at a concentration of 600 mM PPO (Fig. S5), which was similar to that of low concentrations (20–200 mM), indicating that *Pt*TA exhibited good substrate tolerance.

### Kinetic parameters of *Pt*TA

The kinetic parameters of *Pt*TA were investigated (Fig. S6), and the *K*_m_ value was 35.85 mM, which was similar to that of the reported transaminase (Table S3). In addition, the *k*_cat_/*K*_m_ value of *Pt*TA (0.73 S^−1^ mM^−1^) was 2.21-fold and 2.43-fold higher than our previously reported *Pf*TA (0.33 S^−1^ mM^−1^) and *Se*TA (0.30 S^−1^ mM^−1^), respectively (Table S3). The results suggested that *Pt*TA had an obvious advantage in catalytic efficiency towards PPO.

### Asymmetric synthesis of L-PPT from PPO via three-enzyme cascade

For overcoming unfavorable thermodynamic equilibrium, a biocatalytic cascade system was designed by coupling *Pt*TA, GluDH, and GDH (Fig. 1a). An NAD^+^-dependent GluDH from *Lysinibacillus sphaericus* (*Ls*GluDH) was employed to regenerate the amino donor L-Glu. Considering the high cost of NADH, a GDH from *Exiguobacterium sibiricum* (*Es*GDH) was employed for cofactor NADH regeneration.

**Fig. 5:**
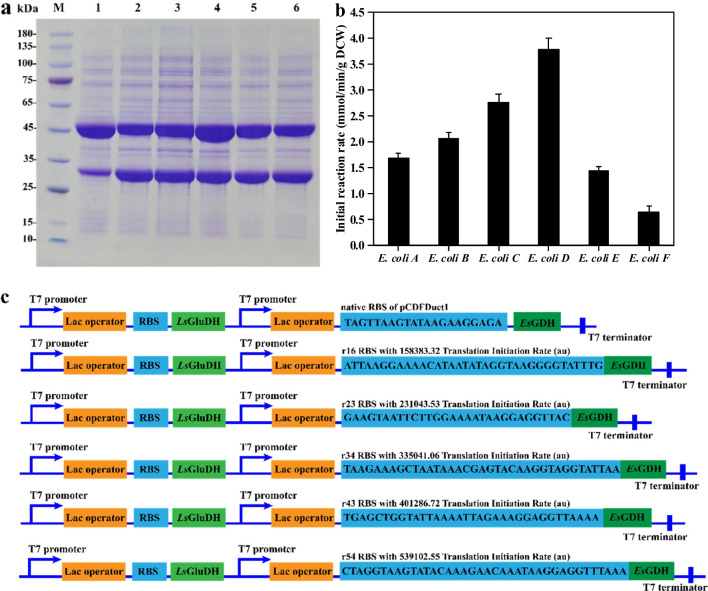
Construction of an in vivo three-enzyme cascade for asymmetric synthesis of L-PPT. **a** SDS-PAGE analysis of crude enzyme for recombinant *E. coli* (A–F). Lane M, protein marker; Lane 1, crude extract of *E. coli* A; Lane 2, crude extract of *E. coli* B; Lane 3, crude extract of *E. coli* C; Lane 4, crude extract of *E. coli* D; Lane 5, crude extract of *E. coli* E; Lane 6, crude extract of *E. coli* F. **b** Initial reaction rates of reduced amination of α-KG to L-Glu by recombinant *E. coli* (A-F) **c** RBS regulation of recombinant plasmids pCDFDUET1-*Ls*GluDH-rbs*Es*GDH

Whole-cell catalysts were more suitable for cofactor-dependent reactions than isolated enzymes (de Carvalho 2011; Wu and Li 2018). To construct an in vivo cascade, the *Ls*GluDH and *Es*GDH were cloned into the plasmid pCDF-Duet1 and transferred into *E. coli* BL21(DE3) for co-expression. The generated recombinant *E. coli* A was analyzed by SDS-PAGE (Fig. 5a), *Ls*GluDH showed an excellent expression level, whereas *Es*GDH expression was low, which might affect the efficiency of cofactor regeneration, further resulting in poor catalytic efficiency of cascade reaction. To strengthen the expression of *Es*GDH, five RBS sequences with different translation initial rates (TIR) were equipped into the plasmid pCDF-Duet1-*Ls*GluDH-*Es*GDH (Fig. 5c), resulting in recombinant *E. coli* (B-F) (Table S2). Analyzed by SDS-PAGE (Fig. 5a), the results indicated that *Es*GDH expression was enhanced. Meanwhile, the *Ls*GluDH expression was affected by the enhanced expression of *Es*GDH. To determine the effects of changes in expression, the efficiency of L-Glu regeneration catalyzed by recombinant *E. coli* (A-F) was measured. As shown in Fig. 5b, the recombinant *E. coli* D containing plasmid pCDF-Duet1-*Ls*GluDH-r34*Es*GDH showed the highest initial reaction rate (3.79 mM^−1^ min^−1^ g^−1^ DCW), which was 2.26-fold higher than that of recombinant *E. coli* A without expression optimization. Therefore, recombinant *E. coli* G that co-expressed *Ls*GluDH, *Es*GDH, and *Pt*TA was generated by co-transferring plasmid pCDF-Duet1-*Ls*GluDH-r34*Es*GDH and plasmid pET28a-*Pt*TA (Table S2).

**Fig. 6:**
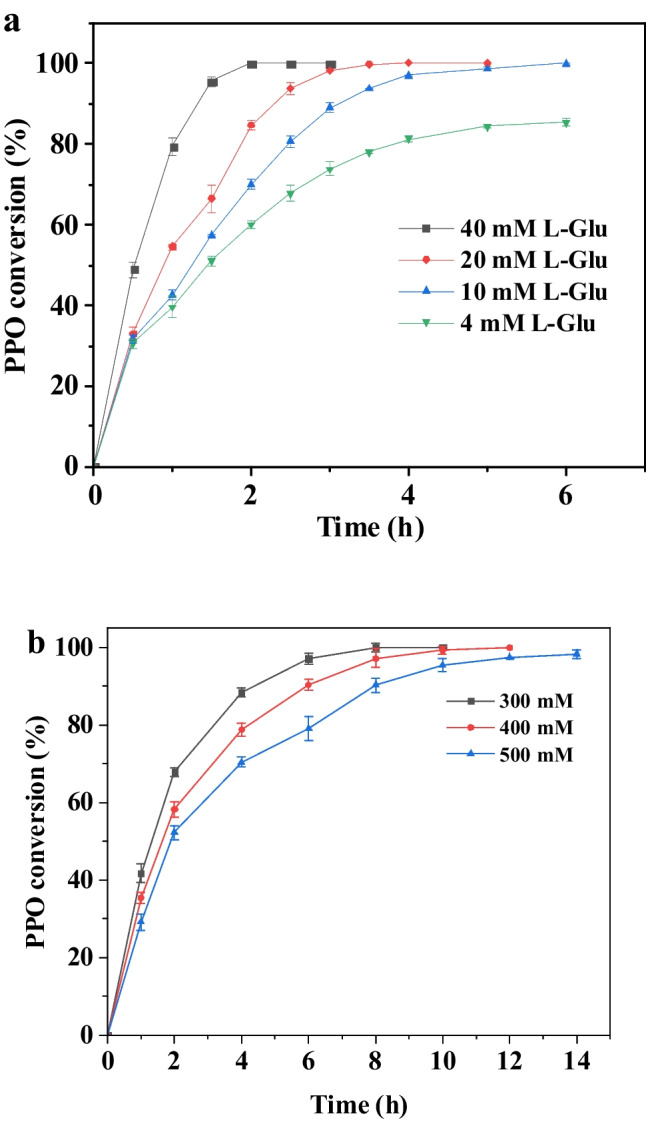
Asymmetric synthesis of L-PPT by recombinant *E. coli* G. **a** Optimization of addition concentrations of amino donor L-Glu. **b** Asymmetric synthesis of L-PPT via enzyme cascade under different substrate concentrations

To demonstrate the availability of in vivo cascade recombinant *E. coli* G for L-PPT production, the reaction was performed using different concentrations of L-Glu (4–40 mM). As shown in Fig. 6a, when 20 mM L-Glu (1:10 molar ratio to PPO) was used, it completely converted 200 mM PPO into L-PPT within 4 h. When the L-Glu concentration was decreased to 10 mM (1:20 molar ratio to PPO) and 4 mM (1:50 molar ratio to PPO), 99.08% and 85.66% conversion were obtained in 6 h, respectively. To ensure a complete PPO conversion at higher concentrations, the molar ratio of L-Glu to PPO was determined as 1:10. Then, the catalytic reaction conditions were further optimized to explore the potential of recombinant *E. coli* G. The effect of pH and temperature on cascade reaction showed that PPO conversion was highest at pH 8.0 and that 40 °C was the optimal reaction temperature (Fig. S7). As is well known, the amino transfer reactions catalyzed by transaminases required the participation of PLP; thus, the concentration of PLP was optimized. As shown in Fig. S8a, 0.1 mM PLP was sufficient to reach maximum conversion. The optimal concentration of the cofactor NAD^+^ for the cascade reaction was 0.1 mM (Fig. S8b). (NH_4_)_2_SO_4_ was used as the amine donor for L-Glu regeneration, and 240 mM (1.2:1 molar ratio to PPO) was determined as the optimal addition concentration (Fig. S8c). Furthermore, 260 mM D-glucose (1.3:1 molar ratio to PPO) was required (Fig. S8d), which was the substrate for *Es*GDH regenerating NADH.

Following the optimal reaction conditions, the cascade reaction was carried out using different PPO concentrations (300–500 mM). As shown in Fig. 6b, PPO could be completely converted to L-PPT within 12 h at concentrations below 400 mM, and a 98.36% conversion was achieved at 500 mM PPO in 14 h.

### One-pot deracemization of D, L-PPT via two-transaminase cascade

One-pot deracemization is probably the most efficient strategy in the biosynthesis of pure enantiomer from the racemic mixture (Han et al. 2019; Parmeggiani et al. 2019). In view of the above considerations, a two-transaminase cascade was designed for L-PPT production from D, L-PPT via one-pot synthesis (Fig. 1b). Based on our previous report (Liu et al. 2023), the *Ym* DAAT exhibited high activity (48.95 U/mg) and affinity (*K*_m_ = 27.49 mM) towards D-PPT. Thus, *Ym* DAAT was employed to catalyze the amino transformation between D-PPT and PPO, followed by L-PPT synthesis catalyzed by *Pt*TA. Through the clever combination of the amino donor (here L-Glu) and *Pt*TA, the resulting by-product (here: α-KG) can serve as the amino acceptor for *Ym* D-AAT. The “two-transaminase cascade,” which combined a linear cascade with a cyclic cascade to reduce simultaneous by-product inhibition of two transaminases, aimed to reduce the thermodynamic limitation.

**Fig. 7:**
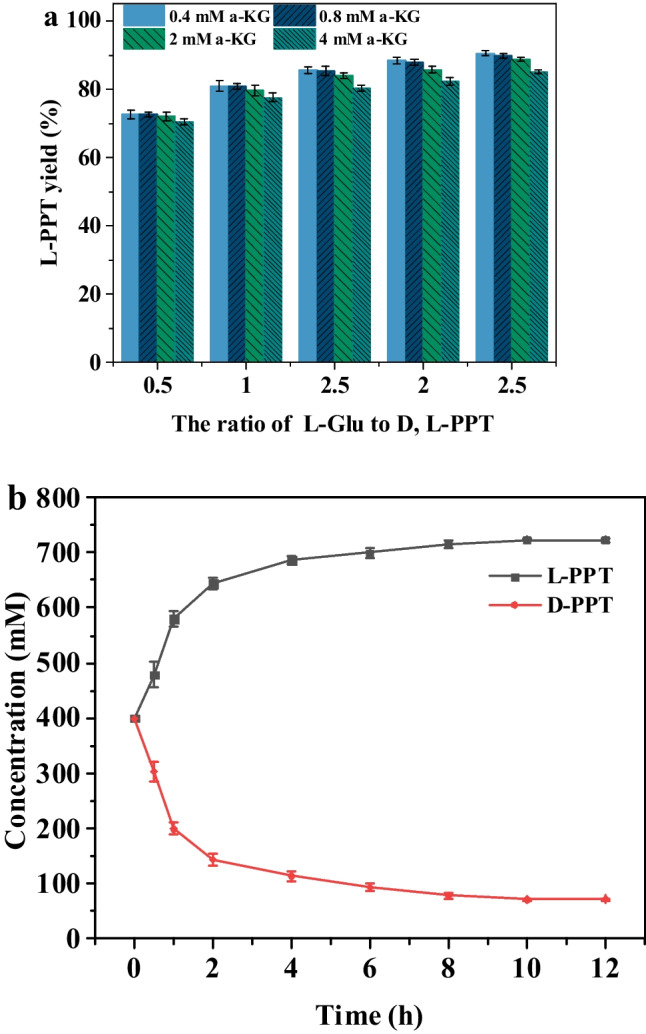
Deracemization of D, L-PPT via two-transaminase cascade. **a** Optimization of concentrations of L-Glu and α-KG. **b** Deracemization of D, L-PPT via two-transaminase cascade at 800 mM D, L-PPT concentration

The deracemization of D, L-PPT was attempted employing 40 mM D, L-PPT as substrate. The biggest effect factor on L-PPT yield was the concentration of L-Glu added. The highest L-PPT yield (90.47%) was observed with 100 mM L-Glu, which had a molar ratio of 2.5:1 to D, L-PPT. In contrast, only 0.4 mM α-KG was needed to initiate the cascade reaction, and higher α-KG concentrations (2–4 mM) resulted in a decrease in L-PPT yield (Fig. 7a). Based on the characterization of *Pt*TA and *Ym* DAAT, pH 8.0 was selected as the optimal pH. As shown in Fig. S9, the optimal PLP concentration and reaction temperature were 0.2 mM and 45 °C, respectively. Considering the activity of *Pt*TA and *Ym* DAAT, the catalyst loading ratio was optimized and selected as 2:1 (Fig. S9c).

Following the optimal reaction conditions, the two-transaminase cascade was evaluated at higher substrate concentrations (200–800 mM). The L-PPT yield above 90% was detected at all measured substrate concentrations (Fig. S10), and the L-PPT yields reached 90.43% at 800 mM D, L-PPT concentration (Fig. 7b), demonstrating superior catalytic efficiency at the highest reported substrate concentration.

## Discussion

Asymmetric synthesis of L-PPT is a highly desirable method, and several transaminases have been utilized for this purpose, including *Pf*TA, *Ck*TA, *Se*TA, and GABA-TA from *Enterobacteriaceae*, (Jia et al. 2019; Jin et al. 2019, 2022; Meng et al. 2018). Nevertheless, there are still some issues that need to be improved before industrial production of L-PPT, such as low activity, poor thermal stability, and thermodynamic limitations. The strategy of mining for thermostable transaminase from the genomes of thermophilic organisms through sequence searching has proven to be an effective and targeted approach (Kelly et al. 2020). Thus, a novel *Pt*TA was identified by this method. As anticipated, *Pt*TA demonstrated an obvious advantage in terms of thermal stability (Table S3) with a half-life of 22.65 h at 55 ℃, as well as high enzymatic activity (28.63 U/mg).

In addition to *Pt*TA, GABA-TA from *Enterobacteriaceae* also exhibited a desirable specific activity towards PPO (> 25 U/mg protein), but its stability was poor with a half-life of 2.8 h at 35 ℃. Interestingly, *Pt*TA was identified as GABA-TA as well, which belongs to the transaminase subgroup II. In contrast to the subgroup III ω-transaminases that have a dual-substrate recognition mechanism (Park et al. 2014; Steffen-Munsberg et al. 2016), the GABA-TA could only recognize the substrates containing carboxyl groups, including dicarboxylic substrates (Liu et al. 2005). As a result, *Pt*TA could not accept IPA as an amino donor (Table 1). The dual-substrate recognition mechanism could be interpreted through the two-binding site model that consisted of a large (L) and a small (S) pocket (Park et al. 2012; Shin and Kim 2002), for which active arginine residue in the (L) pocket forms outward and inward conformations depending on whether the substrate contains carboxyl groups (Han et al. 2015). The active site arginine in GABA-TA is completely conserved and only has an inward conformation; it played the role of recognizing substrate by forming a salt bridge with the α-carboxyl group of incoming substrates (Liu et al. 2005). In addition to the active site arginine (R398), another arginine residue (R141) was present in *Pt*TA. When α-KG was used as an amino acceptor, the R141 residue in GABA-TA was related to stabilizing the γ-carboxyl group away from the α-carbon (Liu et al. 2004). The docking simulation result indicated that PPO was also stabilized in an appropriate position by R141 residue (Fig. S3). Moreover, the mutant *Pt*TA-R141A was created to verify the role of R141 residue, and it lost most of its enzymatic activity towards PPO. Therefore, it was speculated that R141 residue was also conserved in *Pt*TA, which provided the contribution to the substrate PPO binding.

L-Glu was considered an advantageous amino donor for L-PPT synthesis, since the unfavorable thermodynamic equilibrium can be easily circumvented by coupling with GluDH. The reduction amination catalyzed by GluDH was thermodynamically favorable with a high equilibrium constant (Keq = 10^14^–10^18^) (Cheng et al. 2020), which ensured the complete conversion of α-KG to L-Glu. The cascade that coupled GluDH and transaminase had great potential for unnatural amino acid biosynthesis, and several successful applications were developed, such as 1,2-amino alcohols and (*R*)- and (*S*)-phenylglycines (Jung et al. 2023; Liu et al. 2019). By employing this cascade system, 400 mM PPO was completely converted to L-PPT using only 40 mM amino donor L-Glu. The excellent catalytic performance indicated that the challenge of unfavorable thermodynamic equilibrium was efficiently overcome by the cascade system. In addition, a three-enzyme cascade coupling *Pt*TA, GluDH, and ADH from *Rhodococcus ruber* was attempted (Fig. S11a), whose optimal reaction temperature was 50 ℃ (Fig. S11b). The cascade system achieved 99.9% PPO conversion at 300 mM PPO concentration in 12 h (Fig. S11c), further demonstrating the high thermal stability and organic solvent resistance of *Pt*TA. Besides asymmetric synthesis of L-PPT, deracemization of racemic mixtures was another competitive approach for L-PPT biosynthesis (Cao et al. 2020, 2021; Xu et al. 2019; Zhao et al. 2023a). Among these applications, deracemization of D, L-PPT with high atom economy could be a competitive choice, which converted D-PPT to aimed L-PPT by two-step reaction (Cao et al. 2021). For this purpose, the two-transaminase cascade was designed in a one-pot reaction by coupling *Ym* DAAT and *Pt*TA. Compared to the approach that employed DAAO-leucine dehydrogenase cascade system (100 mM D, L-PPT, 80.3% L-PPT yield) (Zhao et al. 2023a), the two-transaminase cascade further simplified technological processes and increased L-PPT yield at an eightfold substrate concentration (800 mM D, L-PPT, 90.43% L-PPT yield). However, the overuse of L-Glu increases the cost and difficulty of product isolation, which may limit its application on an industrial scale. Therefore, further efforts should be focused on the deracemization of D, L-PPT driven by transaminase, and a “one pot, two-step” strategy should be considered.

In conclusion, a novel thermostable transaminase *Pt*TA with excellent activity, stability, and substrate tolerance was mined. The key active site residues of *Pt*TA (R398, R141, I50, K268, and Q242) were identified, among which R141 residue was identified as a conserved residue for the stabilization of substrate PPO. Then, two enzymatic cascade systems driven by *Pt*TA were developed to explore its application in L-PPT production. For asymmetric synthesis of L-PPT from PPO, an in vivo three-enzyme cascade recombinant *E. coli* G was developed by coupling *Pt*TA, *Ls*GluDH, and *Es*GDH, and a complete conversion of 400 mM PPO was achieved. Moreover, a two-transaminase cascade was constructed for deracemization of D, L-PPT in one pot, and a 90.43% L-PPT yield was obtained at the highest reported substrate concentration. These superior catalytic performances demonstrated that the transaminase-driven cascade system has shown great effectiveness in overcoming the thermodynamic limitations for efficient biosynthesis of L-PPT.

## Supplementary Information

Below is the link to the electronic supplementary material.

Supplementary file1 (PDF 1299 KB)

## Data Availability

All data generated or analyzed during this study are included in this article and its supplementary information files.

## References

[CR1] Börner T, Rämisch S, Bartsch S, Vogel A, Adlercreutz P, Grey C (2017) Three in one: temperature, solvent and catalytic stability by engineering the cofactor-binding element of amine transaminase. ChemBioChem 18(15):1482–1486. 10.1002/cbic.201700236 28470825 10.1002/cbic.201700236

[CR2] Cao C, Cheng F, Xue Y, Zheng Y (2020) Efficient synthesis of L-phosphinothricin using a novel aminoacylase mined from *Stenotrophomonas maltophilia*. Enzyme Microb Technol 135:109493. 10.1016/j.enzmictec.2019.109493 32146938 10.1016/j.enzmictec.2019.109493

[CR3] Cao C, Gong H, Dong Y, Li J, Cheng F, Xue Y, Zheng Y (2021) Enzyme cascade for biocatalytic deracemization of D, L-phosphinothricin. J Biotechnol 325:372–379. 10.1016/j.jbiotec.2020.09.024 33007350 10.1016/j.jbiotec.2020.09.024

[CR4] Cerioli L, Planchestainer M, Cassidy J, Tessaro D, Paradisi F (2015) Characterization of a novel amine transaminase from *Halomonas elongata*. J Mol Catal B Enzym 120:141–150. 10.1016/j.molcatb.2015.07.009

[CR5] Chen Y, Yi D, Jiang S, Wei D (2016) Identification of novel thermostable taurine–pyruvate transaminase from *Geobacillus thermodenitrificans* for chiral amine synthesis. Appl Microbiol Biotechnol 100(7):3101–3111. 10.1007/s00253-015-7129-510.1007/s00253-015-7129-5 26577674 10.1007/s00253-015-7129-5

[CR6] Cheng F, Li H, Zhang K, Li Q, Xie D, Xue Y, Zheng Y (2020) Tuning amino acid dehydrogenases with featured sequences for L-phosphinothricin synthesis by reductive amination. J Biotechnol 312:35–43. 10.1016/j.jbiotec.2020.03.001 32135177 10.1016/j.jbiotec.2020.03.001

[CR7] Cheng F, Li J, Zhou S, Liu Q, Jin L, Xue Y, Zheng Y (2021) A single-transaminase-catalyzed biocatalytic cascade for efficient asymmetric synthesis of l-phosphinothricin. ChemBioChem 22(2):345–348. 10.1002/cbic.202000488 32815302 10.1002/cbic.202000488

[CR8] Cheng F, Zhang J, Jiang Z, Wu X, Xue Y, Zheng Y (2022) Development of an NAD(H)-driven biocatalytic system for asymmetric synthesis of chiral amino acids. Adv Synth Catal 364(8):1450–1459. 10.1002/adsc.202101441

[CR9] Dave UC, Kadeppagari R-K (2019) Alanine dehydrogenase and its applications – a review. Crit Rev Biotechnol 39(5):648–664. 10.1080/07388551.2019.1594153 31018703 10.1080/07388551.2019.1594153

[CR10] de Carvalho CCCR (2011) Enzymatic and whole cell catalysis: finding new strategies for old processes. Biotechnol Adv 29(1):75–83. 10.1016/j.biotechadv.2010.09.001 20837129 10.1016/j.biotechadv.2010.09.001

[CR11] Fang Y, Wang J, Ma W, Yang J, Zhang H, Zhao L, Chen S, Zhang S, Hu X, Li Y, Wang X (2020) Rebalancing microbial carbon distribution for L-threonine maximization using a thermal switch system. Metab Eng 61:33–46. 10.1016/j.ymben.2020.01.009 32371091 10.1016/j.ymben.2020.01.009

[CR12] Guo F, Berglund P (2017) Transaminase biocatalysis: optimization and application. Green Chem 19(2):333–360. 10.1039/C6GC02328B

[CR13] Guo X, Wang X, Liu Y, Li Q, Wang J, Liu W, Zhao ZK (2020) Structure-guided design of formate dehydrogenase for regeneration of a non-natural redox cofactor. Chem Eur J 26(70):16611–16615. 10.1002/chem.202003102 32815230 10.1002/chem.202003102

[CR14] Han SW, Park ES, Dong JY, Shin JS (2015) Active-site engineering of ω-transaminase for production of unnatural amino acids carrying a side chain bulkier than an ethyl substituent. Appl Environ Microbiol 81(20):6994–7002. 10.1128/AEM.01533-15 26231640 10.1128/AEM.01533-15 PMC4579442

[CR15] Han SW, Jang Y, Shin JS (2019) In vitro and in vivo one-pot deracemization of chiral amines by reaction pathway control of enantiocomplementary ω-transaminases. ACS Catal 9(8):6945–6954. 10.1021/acscatal.9b01546

[CR16] Hepworth LJ, France SP, Hussain S, Both P, Turner NJ, Flitsch SL (2017) Enzyme cascades in whole cells for the synthesis of chiral cyclic amines. ACS Catal 7(4):2920–2925. 10.1021/acscatal.7b00513

[CR17] Horsman GP, Zechel DL (2017) Phosphonate biochemistry. Chem Rev 117(8):5704–5783. 10.1021/acs.chemrev.6b00536 27787975 10.1021/acs.chemrev.6b00536

[CR18] Jia D, Liu Z, Xu H, Li J, Li J, Jin L, Cheng F, Liu Z, Xue Y, Zheng Y (2019) Asymmetric synthesis of l-phosphinothricin using thermostable alpha-transaminase mined from *Citrobacter koseri*. J Biotechnol 302:10–17. 10.1016/j.jbiotec.2019.06.008 31201835 10.1016/j.jbiotec.2019.06.008

[CR19] Jiang N, Du X, Zheng L (2023) Highly efficient synthesis of chiral lactams by using a ω-transaminase from *Bacillus megaterium* and its mutant enzymes. Mol Catal 547:113364. 10.1016/j.mcat.2023.113364

[CR20] Jin L, Peng F, Liu H, Cheng F, Jia D, Xu J, Liu Z, Xue Y, Zheng Y (2019) Asymmetric biosynthesis of L-phosphinothricin by a novel transaminase from *Pseudomonas fluorescens* ZJB09-108. Process Biochem 85:60–67. 10.1016/j.procbio.2019.07.010

[CR21] Jin L, Shentu J, Liu H, Shao T, Liu Z, Xue Y, Zheng Y (2022) Enhanced catalytic activity of recombinant transaminase by molecular modification to improve L-phosphinothricin production. J Biotechnol 343:7–14. 10.1016/j.jbiotec.2021.11.002 34763007 10.1016/j.jbiotec.2021.11.002

[CR22] Jung DY, Li X, Li Z (2023) Engineering of hydroxymandelate oxidase and cascade reactions for high-yielding conversion of racemic mandelic acids to phenylglyoxylic acids and (R)- and (S)-phenylglycines. ACS Catal 13(2):1522–1532. 10.1021/acscatal.2c05596

[CR23] Kang X, Cai X, Liu Z, Zheng Y (2019) Identification and characterization of an amidase from *Leclercia adecarboxylata* for efficient biosynthesis of L-phosphinothricin. Bioresource Technol 289:121658. 10.1016/j.biortech.2019.121658 10.1016/j.biortech.2019.121658 31234070

[CR24] Kelefiotis-Stratidakis P, Tyrikos-Ergas T, Pavlidis IV (2019) The challenge of using isopropylamine as an amine donor in transaminase catalysed reactions. Org Biomol Chem 17(7):1634–1642. 10.1039/C8OB02342E 30394478 10.1039/c8ob02342e

[CR25] Kelly SA, Mix S, Moody TS, Gilmore BF (2020) Transaminases for industrial biocatalysis: novel enzyme discovery. Appl Microbiol Biotechnol 104(11):4781–4794. 10.1007/s00253-020-10585-0 32300853 10.1007/s00253-020-10585-0 PMC7228992

[CR26] Konia E, Chatzicharalampous K, Drakonaki A, Muenke C, Ermler U, Tsiotis G, Pavlidis IV (2021) Rational engineering of *Luminiphilus syltensis* (R)-selective amine transaminase for the acceptance of bulky substrates. Chem Commun 57(96):12948–12951. 10.1039/D1CC04664K 10.1039/d1cc04664k 34806715

[CR27] Liu W, Peterson PE, Carter RJ, Zhou X, Langston JA, Fisher AJ, Toney MD (2004) Crystal structures of unbound and aminooxyacetate-bound *Escherichia coli* γ-aminobutyrate aminotransferase. Biochemistry US 43(34):10896–10905. 10.1021/bi049218e 10.1021/bi049218e 15323550

[CR28] Liu W, Peterson PE, Langston JA, Jin X, Zhou X, Fisher AJ, Toney MD (2005) Kinetic and crystallographic analysis of active site mutants of *Escherichia coli* γ-aminobutyrate aminotransferase. Biochemistry US 44(8):2982–2992. 10.1021/bi048657a 10.1021/bi048657a 15723541

[CR29] Liu S, Zhang X, Liu F, Xu M, Yang T, Long M, Zhou J, Osire T, Yang S, Rao Z (2019) Designing of a cofactor self-sufficient whole-cell biocatalyst system for production of 1,2-amino alcohols from epoxides. ACS Synth Biol 8(4):734–743. 10.1021/acssynbio.8b00364 30840437 10.1021/acssynbio.8b00364

[CR30] Liu HL, Wu JM, Deng XT, Yu L, Yi PH, Liu ZQ, Xue YP, Jin LQ, Zheng YG (2023) Development of an aminotransferase-driven biocatalytic cascade for deracemization of d, l-phosphinothricin. Biotechnol Bioeng. 10.1002/bit.28432 37227020 10.1002/bit.28432

[CR31] Manaia CM, Moore ERB (2002) Pseudomonas thermotolerans sp. nov., a thermotolerant species of the genus *Pseudomonas sensu stricto*. Int J Syst Evol Micr 52(6):2203–2209 10.1099/00207713-52-6-2203 10.1099/00207713-52-6-2203 12508889

[CR32] Mathew S, Nadarajan SP, Chung T, Park HH, Yun H (2016) Biochemical characterization of thermostable ω-transaminase from *Sphaerobacter thermophilus* and its application for producing aromatic β-and γ-amino acids. Enzyme Microb Technol 87:52–60. 10.1016/j.enzmictec.2016.02.013 27178795 10.1016/j.enzmictec.2016.02.013

[CR33] Mathew S, Renn D, Rueping M (2023) Advances in one-pot chiral amine synthesis enabled by amine transaminase cascades: pushing the boundaries of complexity. ACS Catal 13(8):5584–5598. 10.1021/acscatal.3c00555

[CR34] Meng L, Liu Y, Zhou H, Yin X, Wu J, Wu M, Xu G, Yang L (2018) Driving transamination irreversible by decomposing byproduct α-ketoglutarate into ethylene using ethylene-forming enzyme. Catal Lett 148(11):3309–3314. 10.1007/s10562-018-2552-8

[CR35] Meng Q, Ramírez-Palacios C, Capra N, Hooghwinkel ME, Thallmair S, Rozeboom HJ, Thunnissen A-MWH, Wijma HJ, Marrink SJ, Janssen DB (2021) Computational redesign of an ω-transaminase from *Pseudomonas jessenii* for asymmetric synthesis of enantiopure bulky amines. ACS Catal 11(17):10733–10747. 10.1021/acscatal.1c02053 34504735 10.1021/acscatal.1c02053 PMC8419838

[CR36] Park ES, Kim M, Shin JS (2012) Molecular determinants for substrate selectivity of ω-transaminases. Appl Microbiol Biotechnol 93(6):2425–2435. 10.1007/s00253-011-3584-9 21983703 10.1007/s00253-011-3584-9

[CR37] Park ES, Park SR, Han SW, Dong J-Y, Shin J-S (2014) Structural determinants for the non-canonical substrate specificity of the ω-transaminase from *Paracoccus denitrificans*. Adv Synth Catal 356(1):212–220. 10.1002/adsc.201300786

[CR38] Parmeggiani F, Rué Casamajo A, Walton CJW, Galman JL, Turner NJ, Chica RA (2019) One-pot biocatalytic synthesis of substituted d-tryptophans from indoles enabled by an engineered aminotransferase. ACS Catal 9(4):3482–3486. 10.1021/acscatal.9b00739

[CR39] Petermeier P, Kohlfuerst C, Torvisco A, Fischer RC, Mata A, Dallinger D, Kappe CO, Schrittwieser JH, Kroutil W (2023) Asymmetric synthesis of trisubstituted piperidines via biocatalytic transamination and diastereoselective enamine or imine reduction. Adv Synth Catal 365(13):2188–2202. 10.1002/adsc.202300050

[CR40] Qian W, Ou L, Li C, Pan J, Xu J, Chen Q, Zheng G (2020) Evolution of glucose dehydrogenase for cofactor regeneration in bioredox processes with denaturing agents. ChemBioChem 21(18):2680–2688. 10.1002/cbic.202000196 32324965 10.1002/cbic.202000196

[CR41] Salis HM, Mirsky EA, Voigt CA (2009) Automated design of synthetic ribosome binding sites to control protein expression. Nat Biotechnol 27(10):946–950. 10.1038/nbt.1568 19801975 10.1038/nbt.1568 PMC2782888

[CR42] Shin JS, Kim BG (2002) Exploring the active site of amine:pyruvate aminotransferase on the basis of the substrate structure−reactivity relationship: how the enzyme controls substrate specificity and stereoselectivity. J Org Chem 67(9):2848–2853. 10.1021/jo016115i 11975536 10.1021/jo016115i

[CR43] Slabu I, Galman JL, Lloyd RC, Turner NJ (2017) Discovery, engineering, and synthetic application of transaminase biocatalysts. ACS Catal 7(12):8263–8284. 10.1021/acscatal.7b02686

[CR44] Steffen-Munsberg F, Matzel P, Sowa MA, Berglund P, Bornscheuer UT, Höhne M (2016) *Bacillus anthracis* ω-amino acid:pyruvate transaminase employs a different mechanism for dual substrate recognition than other amine transaminases. Appl Microbiol Biotechnol 100(10):4511–4521. 10.1007/s00253-015-7275-9 26795966 10.1007/s00253-015-7275-9

[CR45] Takano HK, Dayan FE (2020) Glufosinate-ammonium: a review of the current state of knowledge. Pest Manag Sci 76(12):3911–3925. 10.1002/ps.5965 32578317 10.1002/ps.5965

[CR46] Wang J, Zhang H, Yin D, Xu X, Tan T, Lv Y (2021) Boosted activity by engineering the enzyme microenvironment in cascade reaction: a molecular understanding. Syn Syst Biotechnol 6(3):163–172. 10.1016/j.synbio.2021.06.004 10.1016/j.synbio.2021.06.004 PMC8271104 34278014

[CR47] Wu S, Li Z (2018) Whole-cell cascade biotransformations for one-pot multistep organic synthesis. ChemCatChem 10(10):2164–2178. 10.1002/cctc.201701669

[CR48] Wu C, Ma C, Li Q, Chai H, He Y-C (2023) Efficient production of hydroxymethyl-2-furfurylamine by chemoenzymatic cascade catalysis of bread waste in a sustainable approach. Bioresource Technol 385:129454. 10.1016/j.biortech.2023.129454 10.1016/j.biortech.2023.129454 37406829

[CR49] Xie Y, Wang J, Yang L, Wang W, Liu Q, Wang H, Wei D (2022) The identification and application of a robust ω-transaminase with high tolerance towards substrates and isopropylamine from a directed soil metagenome. Catal Sci Technol 12(7):2162–2175. 10.1039/D1CY02032C

[CR50] Xu J, Li F, Xue Y, Zheng Y (2019) Efficient racemization of N-phenylacetyl-D-glufosinate for L-glufosinate production. Chirality 31(7):513–521. 10.1002/chir.23076 31136021 10.1002/chir.23076

[CR51] Zan R, Zhu L, Wu G, Zhang H (2023) Identification of novel peptides with alcohol dehydrogenase (adh) activating ability in chickpea protein hydrolysates. Foods 12(8):1574. 10.3390/foods12081574 37107370 10.3390/foods12081574 PMC10137677

[CR52] Zhang S, Han Y, Kumar A, Gao H, Liu Z, Hu N (2017) Characterization of an L-phosphinothricin resistant glutamine synthetase from Exiguobacterium sp. and its improvement. Appl Microbiol Biotechnol 101(9):3653–3661. 10.1007/s00253-017-8103-1 28175947 10.1007/s00253-017-8103-1

[CR53] Zhao L, Zhang W, Wang Q, Wang H, Gao X, Qin B, Jia X, You S (2023a) A novel NADH-dependent leucine dehydrogenase for multi-step cascade synthesis of L-phosphinothricin. Enzyme Microb Technol 166:110225. 10.1016/j.enzmictec.2023.110225 36921551 10.1016/j.enzmictec.2023.110225

[CR54] Zhao X, Fu K, Xiang K, Wang L, Zhang Y, Luo Y (2023b) Comparison of the chronic and multigenerational toxicity of racemic glufosinate and l-glufosinate to *Caenorhabditis elegans* at environmental concentrations. Chemosphere 316:137863. 10.1016/j.chemosphere.2023.137863 36649895 10.1016/j.chemosphere.2023.137863

[CR55] Zhou H, Meng L, Yin X, Liu Y, Wu J, Xu G, Wu M, Yang L (2020) Biocatalytic asymmetric synthesis of l-phosphinothricin using a one-pot three enzyme system and a continuous substrate fed-batch strategy. Appl Catal A Gen 589:117239. 10.1016/j.apcata.2019.117239

[CR56] Zhu D, Hua L (2009) Biocatalytic asymmetric amination of carbonyl functional groups – a synthetic biology approach to organic chemistry. Biotechnol J 4(10):1420–1431. 10.1002/biot.200900110 19757497 10.1002/biot.200900110

