# Supplementary material for: Identification of a novel thermostable transaminase and its application in L-phosphinothricin biosynthesis
Source: Appl Microbiol Biotechnol. 2024 Jan 30;108:184. doi: 10.1007/s00253-024-13023-7 (PMC10827958; doi:10.1007/s00253-024-13023-7)
Supplement: Supplementary file 1 [file 253_2024_13023_MOESM1_ESM.pdf]

Identification of a novel thermostable transaminase and its application in L-Phosphinothricin biosynthesis

Han-Lin Liu[1,2,3], Pu-Hong Yi[1,2,3], Jia-Min Wu[1,2,3] , Feng-Cheng[1,2,3], Zhi-Qiang Liu[1,2,3], Li-Qun Jin[1,2,3] *, Ya-Ping Xue[1,2,3] , Yu-Guo Zheng[1,2,3]

1 The National and Local Joint Engineering Research Center for Biomanufacturing of Chiral Chemicals, Zhejiang University of Technology, Hangzhou 310014, P. R. China

2 Engineering Research Center of Bioconversion and Biopurification of Ministry of Education, Zhejiang University of Technology, Hangzhou 310014, P. R. China

3 Key Laboratory of Bioorganic Synthesis of Zhejiang Province, College of Biotechnology and Bioengineering, Zhejiang University of Technology, Hangzhou 310014, P. R. China

***Author for correspondence:**

Key Laboratory of Bioorganic Synthesis of Zhejiang Province, College of Biotechnology and Bioengineering, Zhejiang University of Technology, Hangzhou 310014, China

jlq@zjut.edu.cn,

Phone number: +86-571-88320614

Table S1 Strains, plasmids, rbs sequences and primers

Strain, plasmid, or primer	Genotype or sequence	Source
strains		
E. coli BL21 (DE3)	Protein expression host	laboratory store
Pseudomonas thermotolerant	Source of *Pt*TA	WP_017938159.1
Lysinibacillus sphaericus	Source of *Ls*GluDH	PDB:1LEH
Exiguobacterium sibiricum	Source of *Es*GDH	KM817194.1
Bacillus sp. YM-1	Source of *Ym* DAAT	(Liu et al. 2023)
Rhodococcus ruber	Source of ADH	WP 159419335.1
plasmids		
pET28a (+)	Expression vector, T7 promoter; ColE1 ori; Kan^R	laboratory store
pCDFDuet1	Expression vector, T7 promoter; CloDF13 ori; Sm^R	laboratory store
pET28a (+)-*Pt*TA	Kan^R, expression *Pt*TA in *E. coli*	This study
pET28a (+)-*Ym* DAAT	Kan^R, expression *Ym* DAAT in *E. coli*	
pCDFDuet1- *Ls*GluDH-*Es*GDH	Sm^R, co-expression *Ls*GluDH and *Es*GDH in *E. coli*	this study
pCDFDuet1-*Ls*GluDH-r16*Es*GDH	Sm^R, co-expression *Ls*GluDH and *Es*GDH in *E. coli* using a synthetic RBS with 158383.32 TIR	this study
pCDFDuet1-*Ls*GluDH-r23*Es*GDH	Sm^R, co-expression *Ls*GluDH and *Es*GDH in *E. coli* using a synthetic RBS with 231043.53 TIR	this study
pCDFDuet1-*Ls*GluDH-r34*Es*GDH	Sm^R, co-expression *Ls*GluDH and *Es*GDH in *E. coli* using a synthetic RBS with 335041.06 TIR	this study
pCDFDuet1-*Ls*GluDH-r43*Es*GDH	Sm^R, co-expression *Ls*GluDH and *Es*GDH in *E. coli* using a synthetic RBS with 435578.30 TIR	this study
pCDFDuet1-*Ls*GluDH-r54*Es*GDH	Sm^R, co-expression *Ls*GluDH and *Es*GDH in *E. coli* using a synthetic RBS with 539102.55 TIR	this study
RBS	Sequence (5'-3')	
r16*Es*GDH	ATTAAGGAAAACATAATATAGGTAAGGGGTATTTG	this study
r23*Es*GDH	GAAGTAATTCTTGGAAAATAAGGAGGTTAC	this study
r34*Es*GDH	TAAGAAAGCTAATAAACGAGTACAAGGTAGGTATTAA	this study
r43*Es*GDH	TGAGCTGGTATTAAAATTAGAAAGGAGGTTAAAA	this study
r54*Es*GDH	CTAGGTAAGTATACAAAGAACAAATAAGGAGGTTTAAA	this study
primers	Sequence (5'-3')	
L-pET28a-F	CTCGAGCACCACCACCAC	this study
L-pET28a-R	GGTATATCTCCTTCTTAA	this study
A- *Pt*TA-F	TTAAGAAGGAGATATACCATGAGCAAAAACGAAAGC	this study
A- *Pt*TA-R	GTGGTGGTGGTGCTCGAGTTATGCCAGTTCATCAAA	this study
L-pETDuet1-*Ls*GluDH-F	TCGAGTCTGGTAAAGAAACCGCT	this study
L-pETDuet1-*Ls*GluDH-R	TGTATATCTCCTTCTTATACTTAACTAATATACTAAGATGGGGAA	this study
A-*Es*GDH-F	AGAAGGAGATATACAATGGGTTATAATTCTCTGAAAGGCAAAGT	this study
A-*Es*GDH-R	CTTTACCAGACTCGATCAACCACGGCCAGCC	this study
L-rbs-pCDFDuet1-*Ls*GluDH-F	TCGAGTCTGGTAAAGAAACCGCT	this study
L-rbs-pCDFDuet1-*Ls*GluDH-R	ATATACTAAGATGGGGAATTGTTATCCGCT	this study

A-r16*Es*GDH-F	CCCATCTTAGTATATATTAAGGAAAACATAATATAGGTAAGGGGTATTTGATGGGTTATAATTCTCTG	this study
A-r23*Es*GDH-F	CCCATCTTAGTATATGAAGTAATTCTTGGAAAATAAGGAGGTTACATGGGTTATAATTCTCTG	this study
A-r34*Es*GDH-F	CCCATCTTAGTATATTAAGAAAGCTAATAAACGAGTACAAGGTAGGTATTAAATGGGTTATAATTCTC	this study
A-r43*Es*GDH-F	CCCATCTTAGTATATTGAGCTGGTATTAAAATTAGAAAGGAGGTTAAAAATGGGTTATAATTCTCTG	this study
A-r54*Es*GDH-F	CCCATCTTAGTATATCTAGGTAAGTATACAAAGAACAAATAAGGAGGTTTAAA ATGGGTTATAATTCTCTG	this study
A-rbs*Es*GDH-R	CTTTACCAGACTCGATCAACCACGGCCAGCC	this study
R141A-F	TTACCGGTGCATATCATGGTCGTACCATGATGACCCTGAGCC	this study
R141A-R	AATGGCCACGTATAGTACCAGCATGGTACTACTGGGACTCGG	this study

Table S2 Construction of co-expression Recombinant *E. coli*

Strain[a]	Recombinant plasmids in the strain	Reaction
E. coli A	pCDFDuet1- *Ls*GluDH-*Es*GDH	α-KG to L-Glu
E. coli B	pCDFDuet1-*Ls*GluDH- r16*Es*GDH	α-KG to L-Glu
E. coli C	pCDFDuet1-*Ls*GluDH- r23*Es*GDH	α-KG to L-Glu
E. coli D	pCDFDuet1-*Ls*GluDH- r34*Es*GDH	α-KG to L-Glu
E. coli E	pCDFDuet1-*Ls*GluDH- r43*Es*GDH	α-KG to L-Glu
E. coli F	pCDFDuet1-*Ls*GluDH- r54*Es*GDH	α-KG to L-Glu
E. coli G	pCDFDuet1-*Ls*GluDH- r34*Es*GDH + pET28a (+)-*Pt*TA	Asymmetric synthesis of L-PPT

*Note: Ls*GluDH (glutamate dehydrogenase form *Lysinibacillus sphaericus*); *Es*GDH (D-glucose dehydrogenease from *Exiguobacterium sibiricum*); *Pt*TA (transaminase from *Pseudomonas thermotolerant*).

Abbreviation:α-KG, α-ketoglutarate; L-Glu, L-glutamate; L-PPT, L-phosphinothricin.

Table S3 Transaminases for asymmetric of L-PPT from PPO

Enzyme	Source	Amino donor	Specific activity (U/mg)	K_m (mM)	K_{cat}/K_m($S^{-1}mM^{-1}$)	Thermal Stability(°C)	Reference
*Pt*TA	*Pseudomonas thermotolerant*	L-Glu	28.63	35.85	0.73	$t_{1/2}$= 22.65 h (55 °C)<br>83.21 % residual activity (55 °C, 6h)	This study
*Ck*TA	*Citrobacter koseri*	L-Glu	4.0	36.75	0.57	65.9 % residual activity (57 °C, 4h)	(Jia et al. 2019)
*Ct*TA	*Cronobacter turicensis*	L-Glu	2.52	55.05	0.49	58.1% residual activity (52 °C, 4h)	(Jia et al. 2019)
*Pf*TA	*Pseudomonas fluorescens*	L-Ala	1.29	34.02	0.33	$t_{1/2}$= 15.38 h (40 °C)	(Jin et al. 2019)
*Se*TA	*Salmonella enterica*	L-Ala	1.74	40.13	0.30	$t_{1/2}$= 4.21 h (50 °C)	(Jin et al. 2022)
GABA-TA	*Enterobacteriaceae*	L-Glu	41.8	N.D	N.D	$t_{1/2}$=2.8 h (35 °C)	(Zhou et al. 2020)

Abbreviation: L-PPT, L-phosphinothricin; PPO, 2-oxo-4-[(hydroxy)(methyl)phosphinoyl]butyric acid; L-Glu, L-glutamate; L-Ala, L-alanine.

a

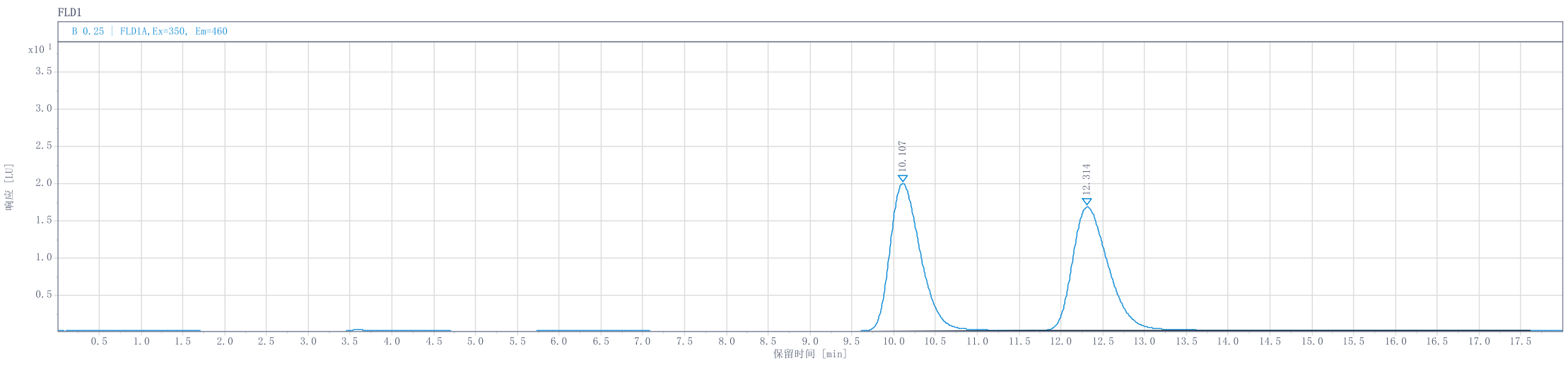

b

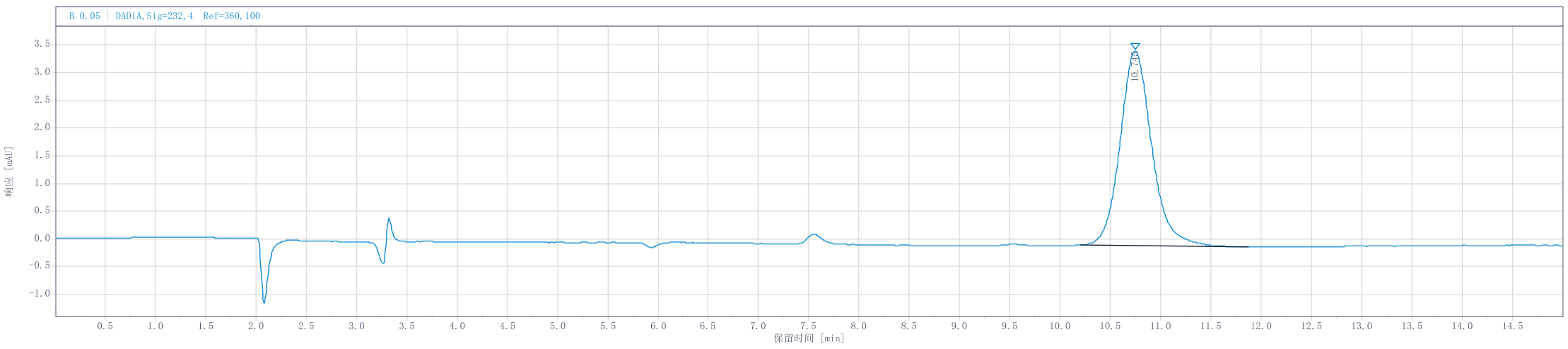

Fig. S1 HPLC chromatograms of individual authentic compounds. (a) the *O*-phthalaldehyde and *N*-acetyl-L-cysteine were used as chiral derivatization reagent for the derivatization of samples (30 °C, 5 min). L-PPT (9.6-11.2 min), D-PPT (11.7-13.3 min). (b) the sampless were directly detected by HPLC without derivatization. PPO (10.2-11.8 min).

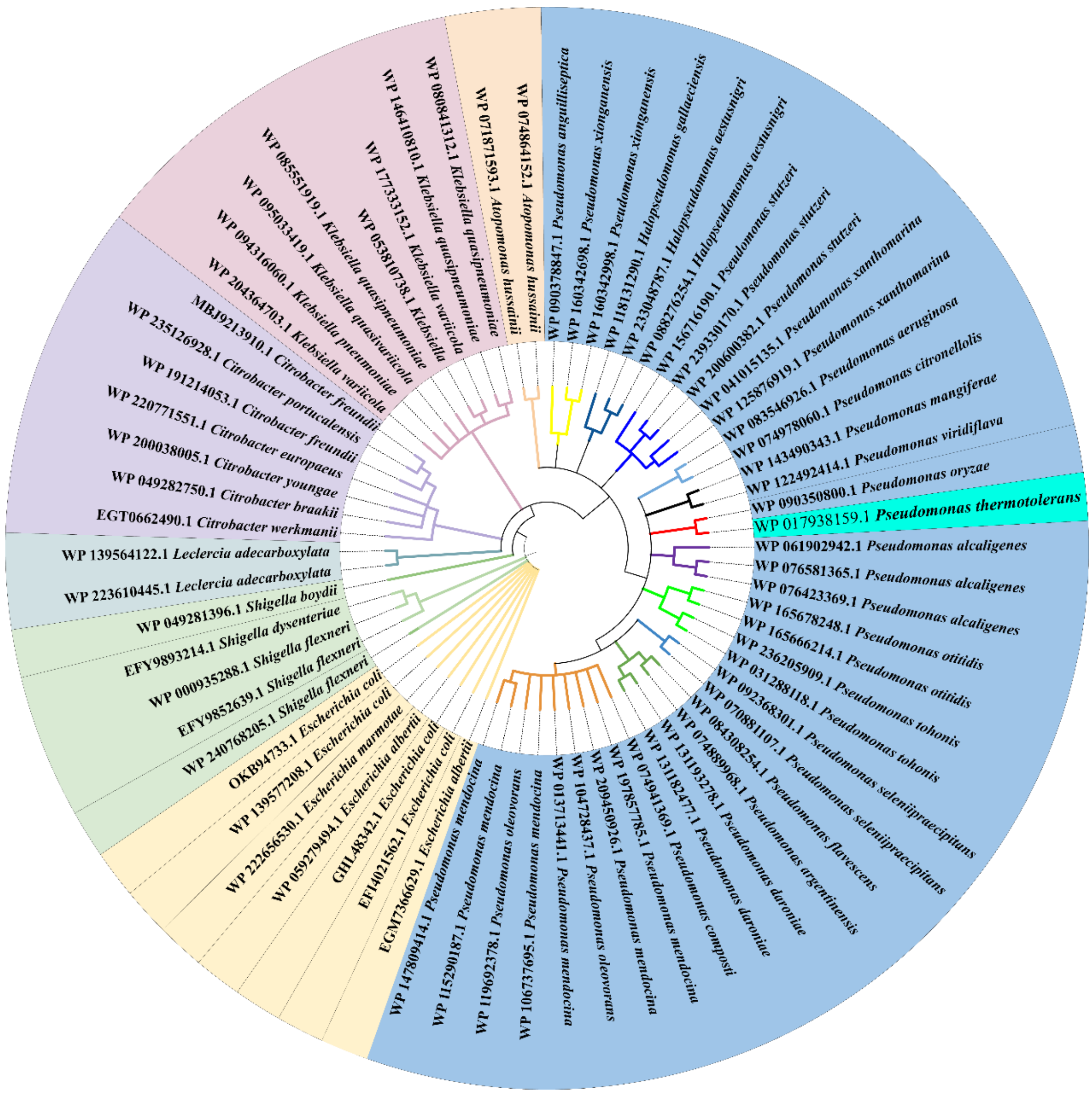

Fig. S2 Phylogenetic tree analysis for searching transaminase gene belonging to thermophilic organisms. The phylogenetic tree was constructed by MEGA 7.0 program and Interaction Tree of Life online tool (https://itol.embl.de/#).

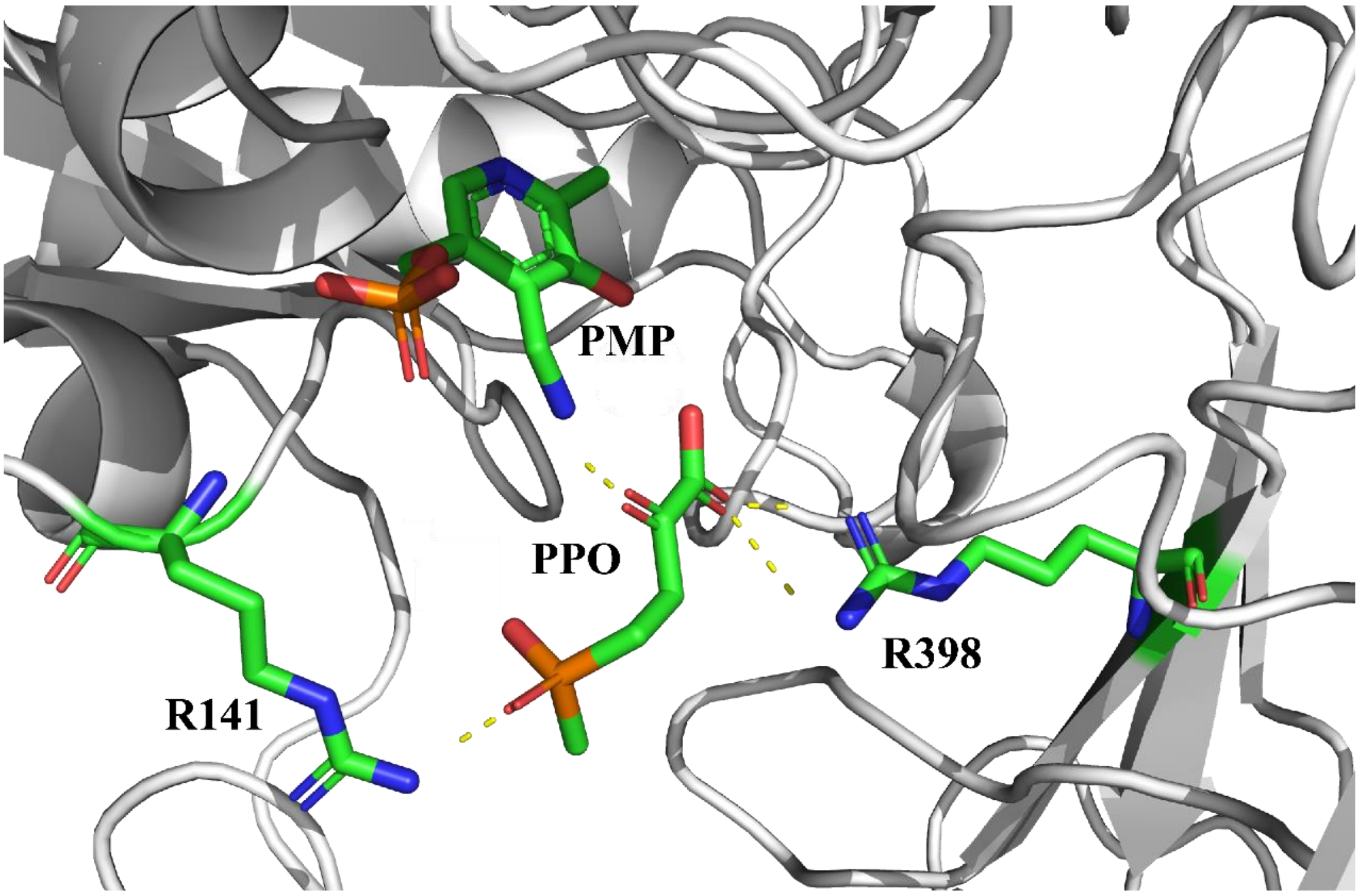

Fig. S3 Substrate docking simulation. Molecule docking simulation was performed using AutoDock 4.2, and the visualization of the homology model with docking simulation was performed by Pymol software. Abbreviation: PMP, pyridoxamine 5′-phosphate; PPO, 2-oxo-4-[(hydroxy)(methyl)phosphinoyl]butyric acid.

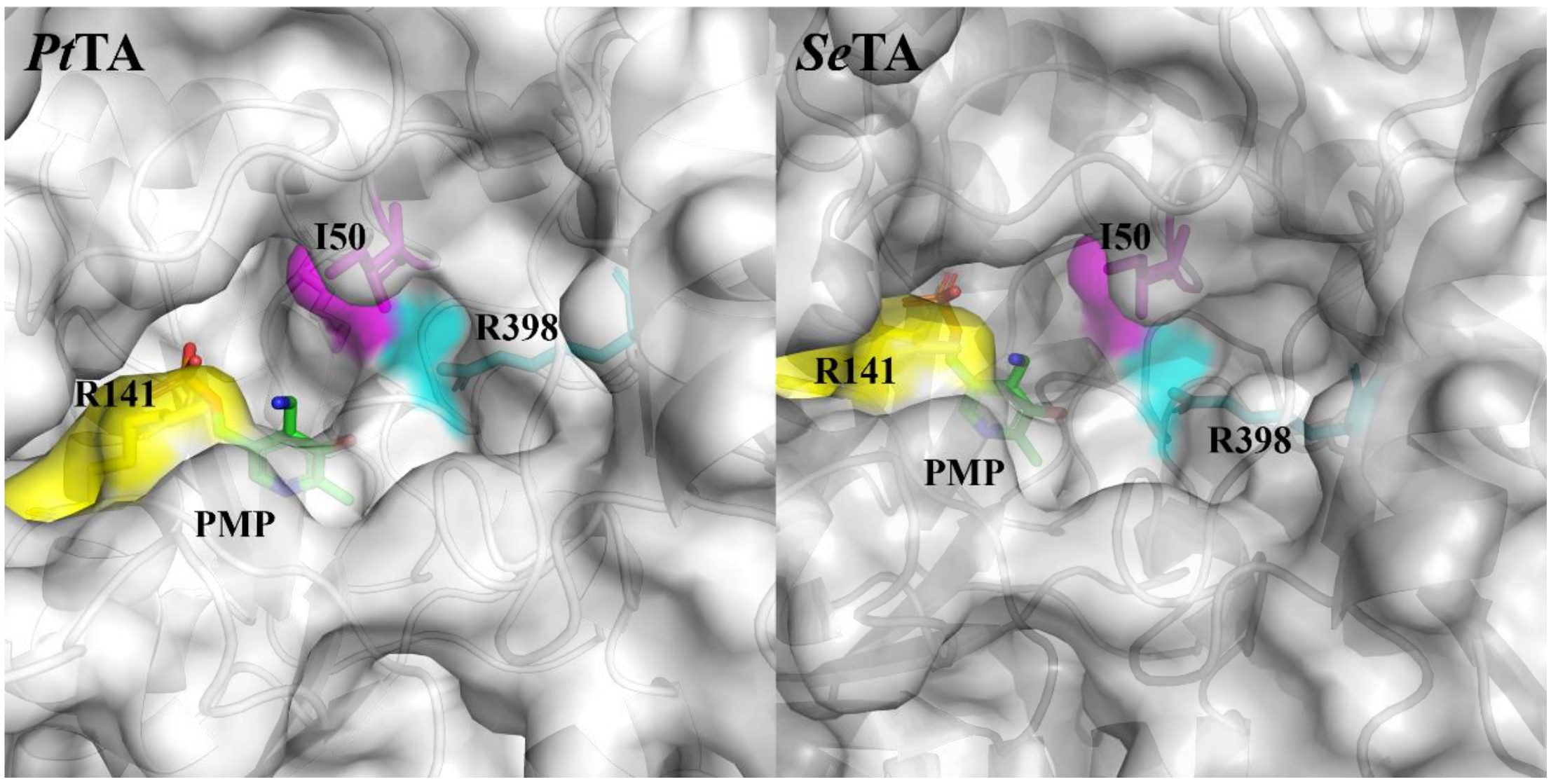

Fig. S4 Substrate binding pocket of *Pt*TA and *Se*TA. Homology modeling of *Pt*TA and *Se*TA was performed by SWISS-MODEL ((https://swissmodel.expasy.org/), the GABA-TA (PDB ID: 1SZK) was used as template. In the models of *Pt*TA and *Se*TA, a cavity above the coenzyme PMP binding site contained key residues I50, R141 and R398. These residues formed substrate binding pocket. Abbreviation: PMP, pyridoxamine 5′-phosphate.

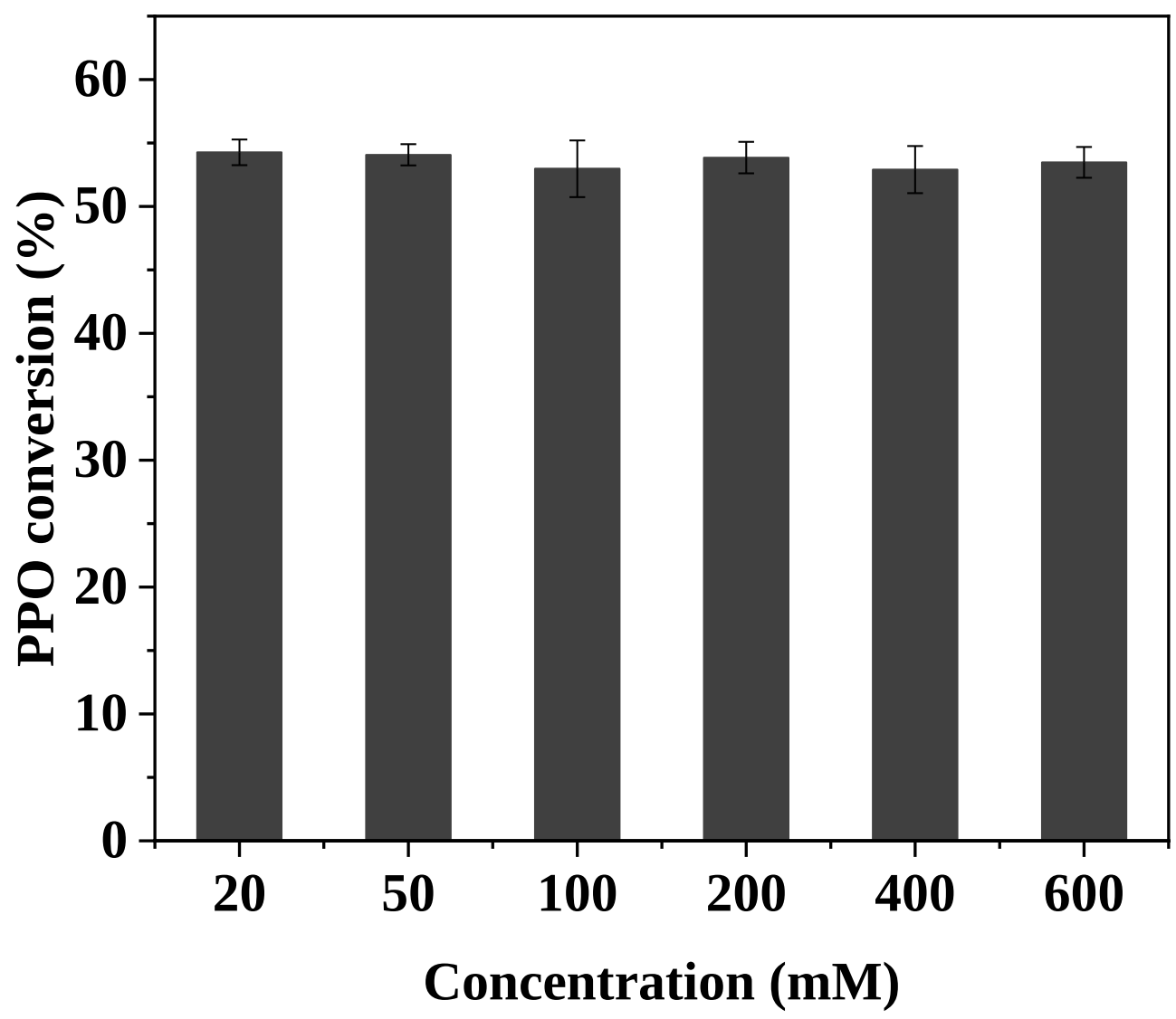

Fig. S5 Effect of substrate concentrations on PPO conversion. Reaction conditions: 20-600 mM PPO, 20-600 mM L-Glutamate (1/1 molar ratio to substrate PPO), 0.1 mM PLP, 2 g L^{-1} DCW *E.coli*/pET28a-*Pt*TA, 10 mL PB buffer (pH 8.0, 50 mM) at 55 ℃, 10 mL for 12h.

Abbreviation: PPO, 2-oxo-4-[(hydroxy)(methyl)phosphinoyl]butyric acid.

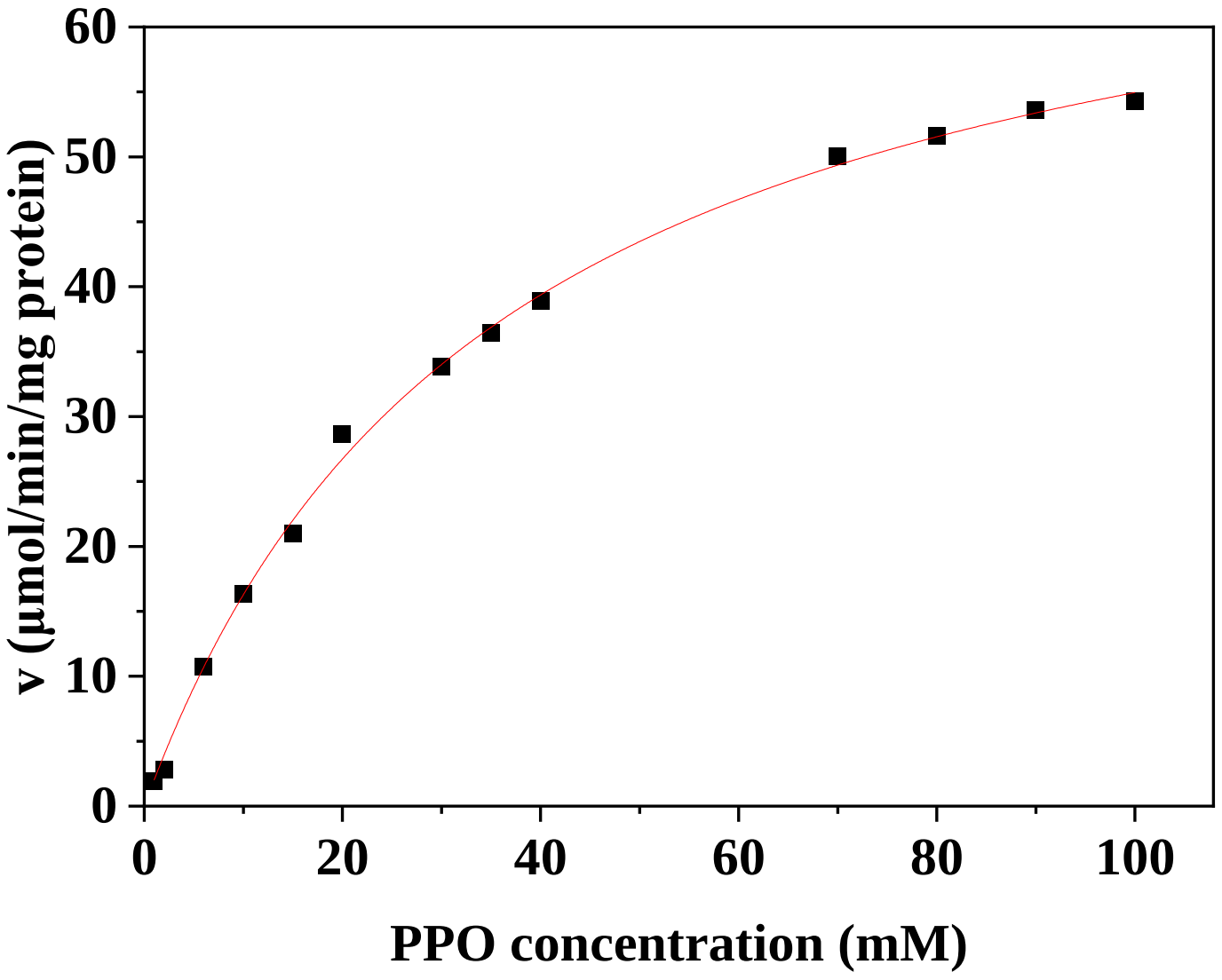

Fig. S6 Nonlinear fit of the Michaelis-Menten model for *Pt*TA. The kinetic parameters of *Pt*TA were measured by non-linear fitting of the Michaelis-Menten model using OriginPro 2023b software. The initial reaction rates of *Pt*TA were measured under the conditions of the enzyme activity assay, except that different concentrations of PPO (1-100 mM) were used as amino acceptor. For the measurement of the initial reaction rate, the substrate conversion was limited to 10%.

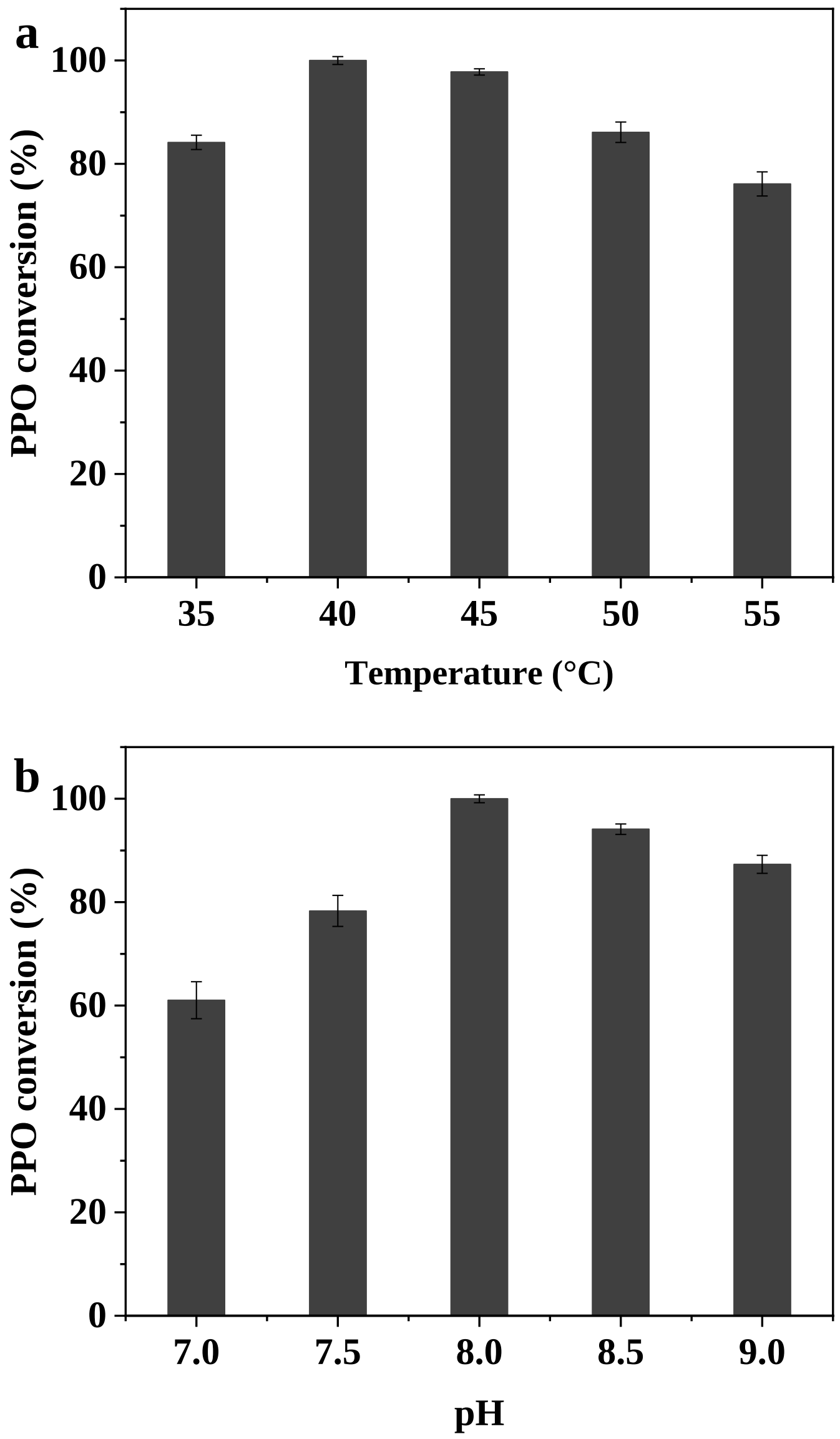

Fig. S7 Effect of pH and temperature on cascade reaction for asymmetric synthesis of L-PPT. **a** Effect of pH on PPO conversion. **b** Effect of temperature on PPO conversion. Reaction conditions: 200 mM PPO, 20 mM L-Glu, 0.1 mM PLP, 0.1 mM NAD^+, 300 mM $(NH_4)_2SO_4$ and 300 mM D-glucose, 2 g L^{-1} DCW *E.coli* G, 30 mL PB buffer (pH 7.0-8.0, 50 mM)/Tris-HCl buff (pH 8.5-9.0, 50 mM) at 35-55 ℃ for 6h. The pH of 30 mL reaction system was controlled by an automatic pH titrator (Metrohm 902 Titrando) by the addition of ammonia.

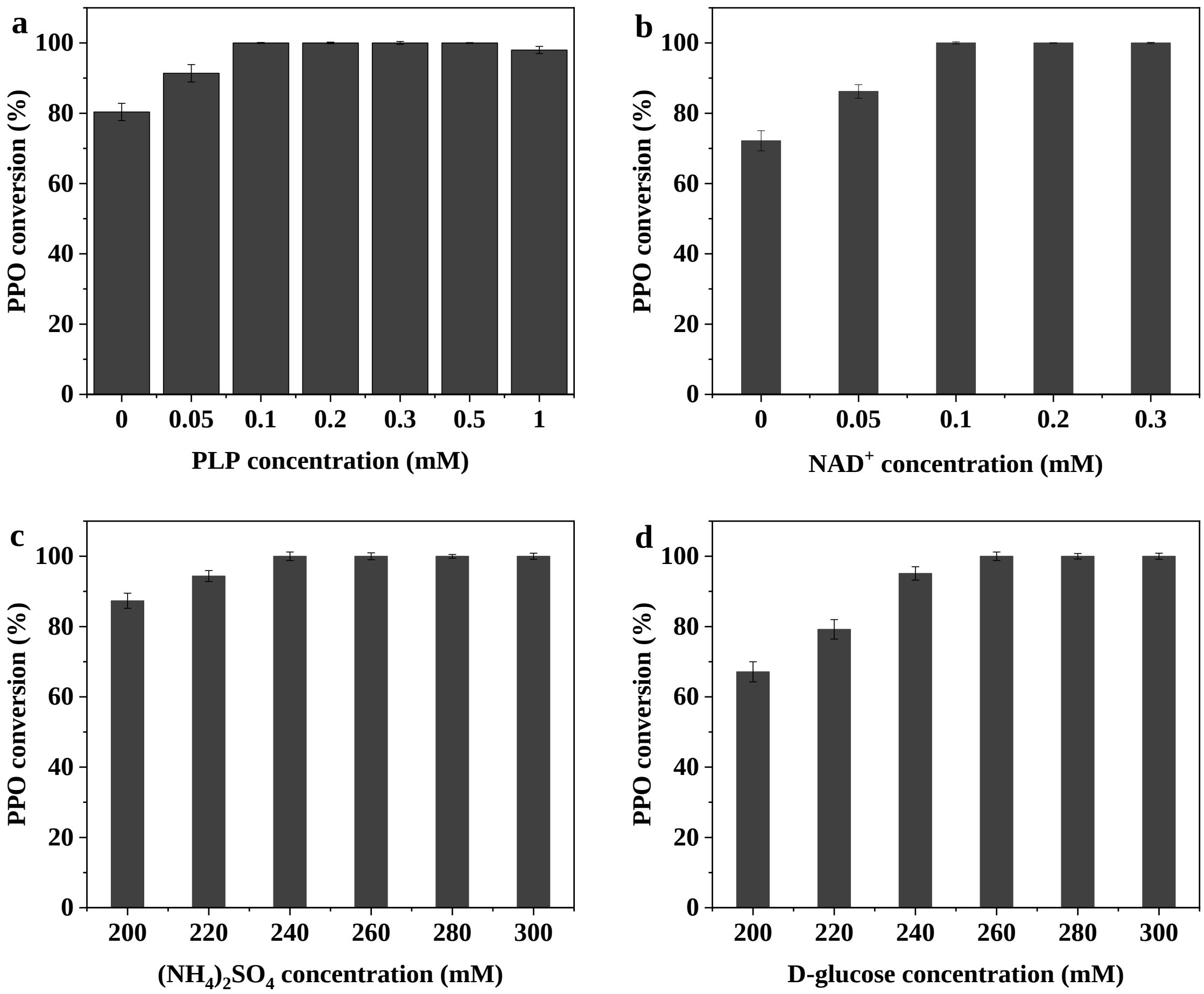

Fig. S8 Optimization of cascade reaction conditions for asymmetric synthesis of L-PPT. **a** Effect of PLP concentration on PPO conversion. b Effect of NAD^+ concentration on PPO conversion. c Effect of $(NH_4)_2SO_4$ concentration on PPO conversion. d Effect of D-glucose concentration on PPO conversion. Reaction conditions: 200 mM PPO, 20 mM L-Glu, 0-1 mM PLP, 0-0.3 mM NAD^+, 200-300 mM $(NH_4)_2SO_4$ and 200-300 mM D-glucose, 2 g L^{-1} DCW *E.coli* G, 30 mL PB buffer (pH 8.0, 50 mM) at 40 ℃ for 6h. The pH of 30 mL reaction system was controlled by an automatic pH titrator (Metrohm 902 Titrando) via the addition of ammonia. For the measurement of initial reaction rate, the substrate conversion was limited to 10%.

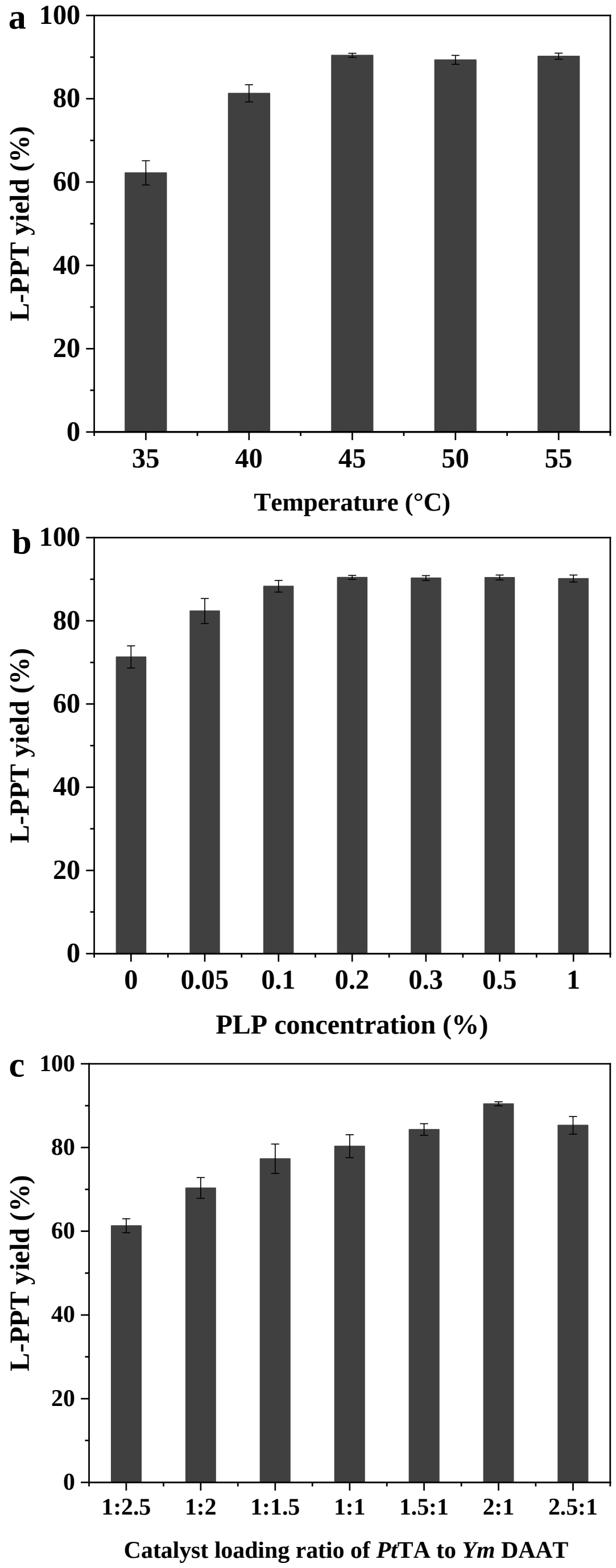

Fig. S9 Optimization of cascade reaction conditions for deracemization of D, L-PPT. **a** Effect of temperature on L-PPT yield. **b** Effect of PLP concentration on L-PPT yield. **c** Effect of the ratio of the catalyst loading on L-PPT yield. Reaction conditions: 10 mL reaction mixtures (PB buffer, 50 mM, pH 8.0) at 35-55 °C for 30 min, containing 40 mM D, L-PPT, 0.4 mM α-KG, 100 mM L-Glu, 0-1 mM PLP, 0.5-2.5 g/L DCW *E. coli* /pET28a-*Pt*TA and 0.5-2.5 g/L *E. coli* /pET28a-*Ym* DAAT.

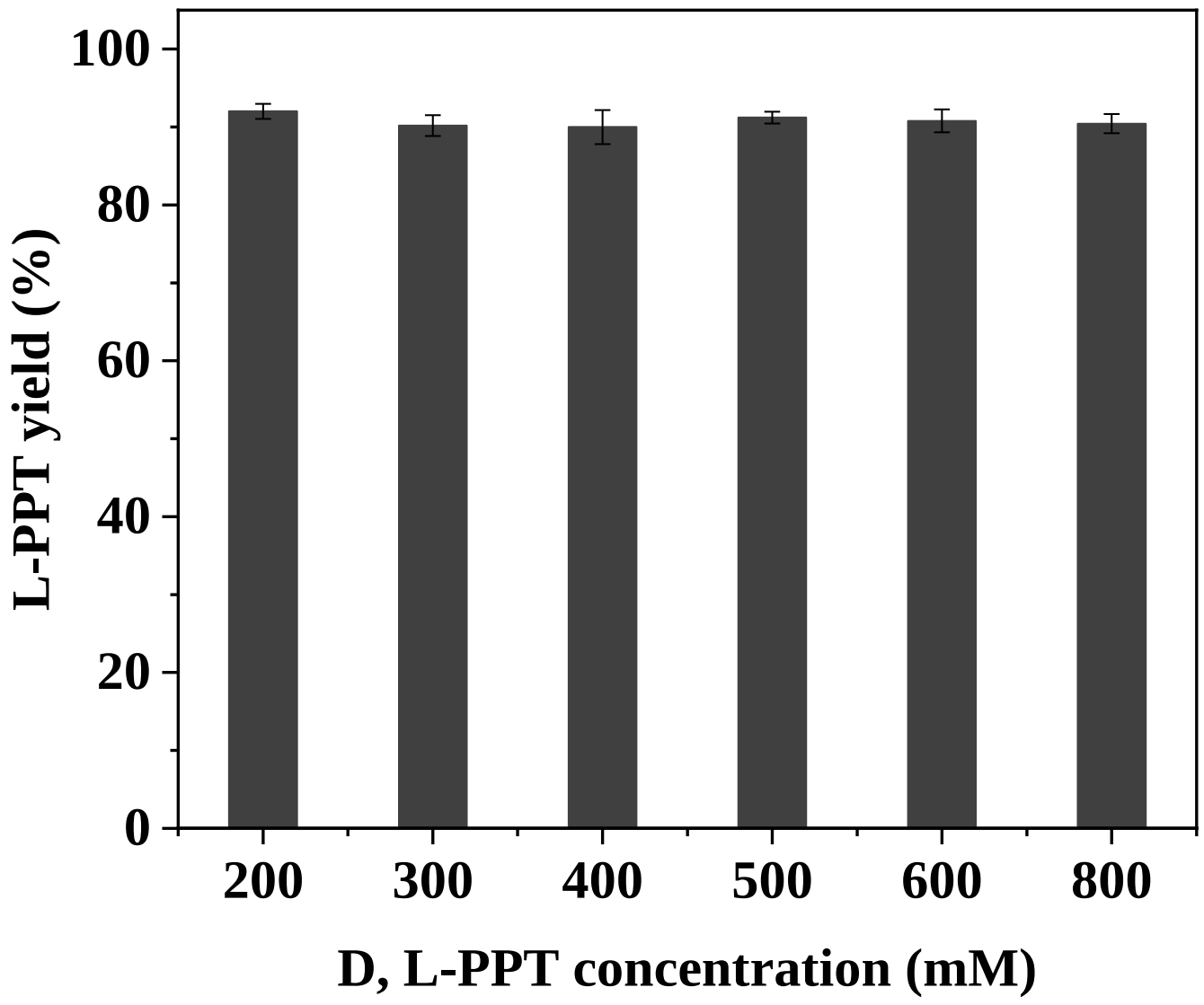

91
92 **Fig. S10** Effect of substrate concentrations on L-PPT yield. Reaction conditions: 200-800 mM D,
93 L-PPT, 2-8 mM α-KG, 0.5-2 M L-Glu, 0.2 mM PLP, 3 g/L DCW *E. coli* /pET28a-*Pt*TA , 1.5 g/L
94 DCW *E. coli* /pET28a-*Ym* DAAT and 30 mL PB buffer (pH 8.0, 50 mM) at 45 °C for 12 h.

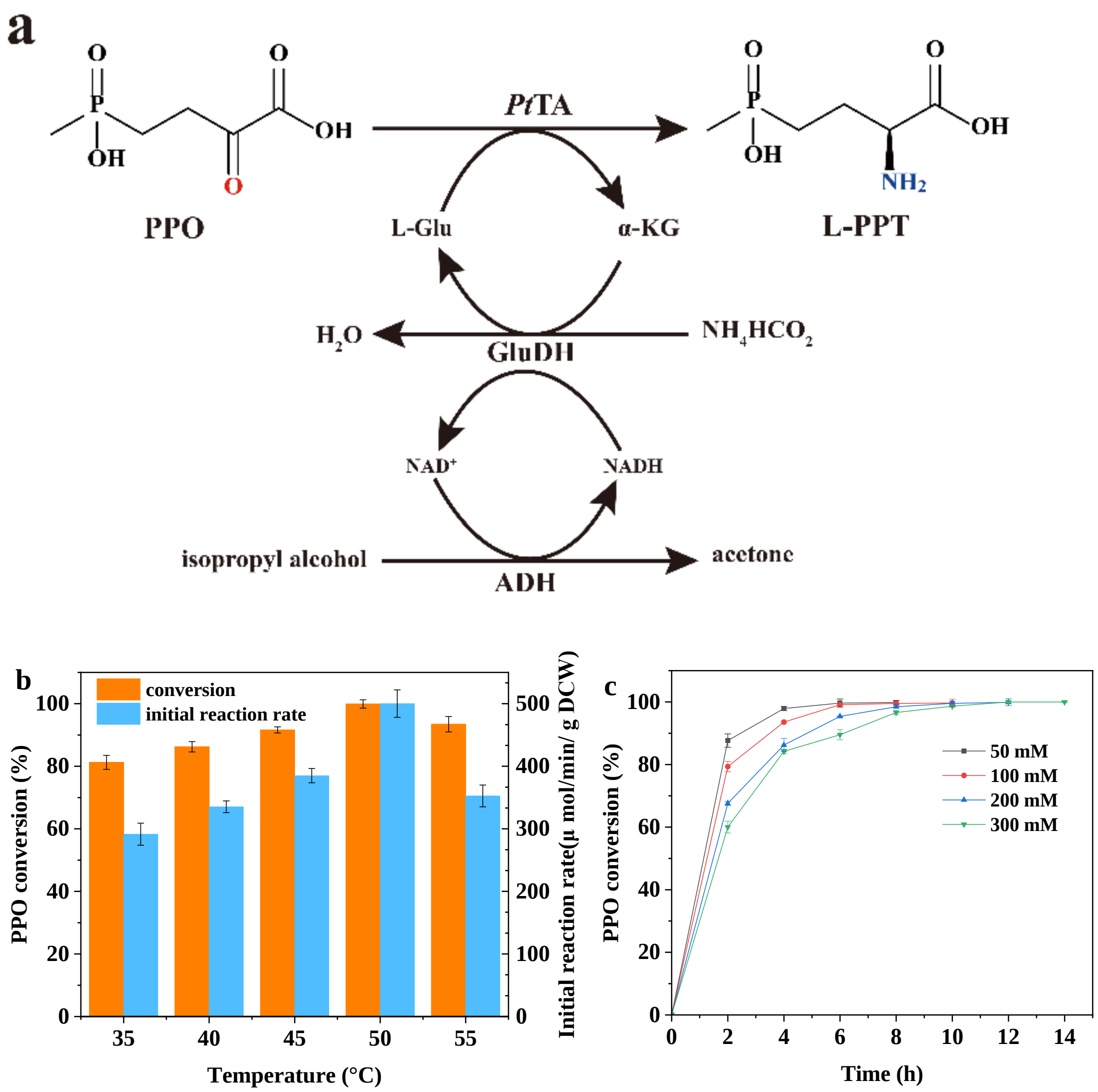

Fig. S11 Three-enzyme cascade coupling *Pt*TA, GluDH and ADH for asymmetric of L-PPT. **a** Three-enzyme cascade coupling *Pt*TA, GluDH and ADH. *Pt*TA (transaminase from *Pseudomonas thermotolerant*); GluDH (glutamate dehydrogenase form *Lysinibacillus sphaericus*); ADH (alcohol dehydrogenase from *Rhodococcus ruber*). **b** Effect of temperature on PPO conversion and initial reaction rate. Reaction condition: 50 mM PPO, 5 mM L-Glu, 0.1 mM PLP, 0.1 mM NAD^+, 75 mM $(NH_4)_2SO_4$ and 75 mM isopropyl alcohol, 2 g L^{-1} DCW *E.coli*/pET28a-*Pt*TA-GluDH-ADH, 30 mL PB buffer (pH 8.0, 50 mM) at 35-55 °C for 8h. For the measurement of initial reaction rate, the substrate conversion was limited to 10%. **c** Asymmetric synthesis of L-PPT via three-enzyme cascade under different PPO concentrations. Reaction condition: 50-300 mM PPO, 5-30 mM L-Glu, 0.1 mM PLP, 0.1 mM NAD+, 75-450 mM (NH4)$_2$SO$_4$ and 75-450 mM isopropyl alcohol, 2 g L^{-1} DCW *E.coli*/pET28a-PtTA-GluDH-ADH, 30 mL PB buffer (pH 8.0, 50 mM) at 50 °C for 14 h.

REFERENCES

Jia D, Liu Z, Xu H, Li J, Li J, Jin L, Cheng F, Liu Z, Xue Y, Zheng Y (2019) Asymmetric synthesis of l-phosphinothricin using thermostable alpha-transaminase mined from *Citrobacter koseri*. J Biotechnol 302:10-17 doi:https://doi.org/10.1016/j.jbiotec.2019.06.008

Jin L, Peng F, Liu H, Cheng F, Jia D, Xu J, Liu Z, Xue Y, Zheng Y (2019) Asymmetric biosynthesis of L-phosphinothricin by a novel transaminase from *Pseudomonas fluorescens* ZJB09-108. Process Biochem 85:60-67 doi:https://doi.org/10.1016/j.procbio.2019.07.010

Jin L, Shentu J, Liu H, Shao T, Liu Z, Xue Y, Zheng Y (2022) Enhanced catalytic activity of recombinant transaminase by molecular modification to improve L-phosphinothricin production. J Biotechnol 343:7-14 doi:https://doi.org/10.1016/j.jbiotec.2021.11.002

Liu HL, Wu JM, Deng XT, Yu L, Yi PH, Liu ZQ, Xue YP, Jin LQ, Zheng YG (2023) Development of an aminotransferase-driven biocatalytic cascade for deracemization of d,l-phosphinothricin. Biotechnol Bioeng doi:https://doi.org/10.1002/bit.28432

Zhou H, Meng L, Yin X, Liu Y, Wu J, Xu G, Wu M, Yang L (2020) Biocatalytic asymmetric synthesis of l-phosphinothricin using a one-pot three enzyme system and a continuous substrate fed-batch strategy. Appl Catal A Gen 589:117239 doi:https://doi.org/10.1016/j.apcata.2019.117239
